# Immune Predictors of Radiotherapy Outcomes in Cervical Cancer

**DOI:** 10.1002/advs.202509784

**Published:** 2026-01-21

**Authors:** Linghao Wang, Jie Zhu, Zequn Ding, Zhiyuan Xie, Xingchen Liu, Feihong Zhang, Xiaojun Liu, Yan Zhang, Haiyan Chen

**Affiliations:** ^1^ Med‐X Research Institute & School of Biomedical Engineering Shanghai Jiao Tong University Shanghai China; ^2^ Department of radiation oncology Ren Ji Hospital School of Medicine Shanghai Jiao Tong University Shanghai China; ^3^ Department of Obstetrics and Gynecology Ren Ji Hospital School of Medicine Shanghai Jiao Tong University Shanghai China; ^4^ Department of Gynecology and Obstetrics The Second Affiliated Hospital of Naval Medical University Shanghai China

**Keywords:** cervical cancer, complement pathways, machine learning, macrophage polarization, radiotherapy outcomes

## Abstract

The immune microenvironment influences the sensitivity of patients to radiotherapy (RT), yet determinants of therapeutic resistance remain elusive. This study integrates single‐cell transcriptomics and machine learning to delineate immune predictors of RT outcomes. Comprehensive analysis reveals reduced epithelial cell numbers, accompanied by enhanced apoptosis, complement activation, and inflammatory responses. RT triggers macrophage accumulation, particularly an RT‐responsive M1‐like HSPA1B+ subset with elevated antigen‐presenting capacity. While T and NK cell cytotoxicity increases, their exhaustion markers (e.g., PDCD1, TIGIT) are exacerbated. CellChat analysis identifies robust epithelial‐myeloid crosstalk mediated by the C3/C3AR1 axis. In murine models, C3AR1 antagonism diminishes RT efficacy, impairing macrophage infiltration and M1 polarization. Leveraging 25 single‐cell‐derived immune features, an 8‐feature multilayer perceptron model: Cervical Cancer Radiotherapy Immune‐Response Model (CCRTIM) is developed. CCRTIM robustly predicts prognosis (AUC = 0.76) and exhibits risk stratification. These findings unveil dynamic immune remodeling post‐RT and establish actionable biomarkers for precision radiotherapy strategies.

## Graphical abstract

This study reveals dynamic immune remodeling in cervical cancer following radiotherapy. Single‐cell analysis identifies the C3/C3AR1 axis as a central mediator of epithelial–myeloid crosstalk, whose inhibition reduces treatment efficacy in mice. Guided by these insights, the eight‐feature machine‐learning model: Cervical Cancer Radiotherapy Immune‐Response Model (CCRTIM) is developed to robustly predict patient prognosis and inform precision immuno‐radiotherapy strategies.

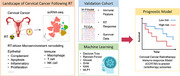

## 1 Introduction

Cervical cancer remains a leading cause of cancer‐related mortality in women globally, with over 661 021 new cases and 348 189 deaths annually, particularly in low‐ and middle‐income countries [1]. Radiotherapy (RT) is the primary treatment modality for locally advanced cervical carcinoma (FIGO 2018 stage IB3–IVA) [2], demonstrating 5‐year overall survival rates ranging from 50% to 60% [3, 4]. Nevertheless, radiotherapy fails in approximately 30% of cervical cancer patients due to intrinsic radioresistance or post‐treatment recurrence [5]. The mechanisms underlying radioresistance are multifactorial, including epigenetic dysregulation, DNA repair pathway activation, and tumor metabolic reprogramming [6, 7]. Recentiy, the tumor immune microenvironment (TIME) emerges as a key regulator of RT response, with mechanistic evidence delineating its modulatory functions [8, 9, 10].

RT exerts dual immunomodulatory effects through regulated mechanisms: the acute phase induces immunogenic cell death, enhancing antigen presentation and pro‐inflammatory effector cell recruitment [11]; while delayed metabolic adaptation generates hypoxic niche‐driven immunosuppression via MDSC and Treg infiltration [12, 13, 14]. Emerging evidence in cervical cancer indicates that RT modulates immune cell subset composition and expression of immunosuppressive molecules including PD‐1 [15]. Single‐cell transcriptomic analyses have further revealed radiation‐induced upregulation of MHC class II molecules and activation of innate immune pathways [16]. However, the specific immune cell populations and molecular determinants that correlate strongly with radiosensitivity and exert positive prognostic influences remain poorly characterized. Elucidating these spatiotemporal immune dynamics represents a critical step toward overcoming therapeutic resistance and developing personalized RT strategies.

Machine learning has emerged as a transformative tool for prognostic prediction, enabling systematic integration of multi‐omics data (radiomic, genomic, and transcriptomic profiles) to elucidate complex immune signatures. Significant advances have been achieved in predicting immune checkpoint blockade (ICB) outcomes through machine learning approaches [17, 18, 19]. Notably, Chang et al. developed a six‐feature logistic regression classifier incorporating clinical‐pathological characteristics to accurately predict ICB responsiveness (AUC = 0.76) [19]. Concurrently, Liu et al. established a predictive framework utilizing macromolecular biomarkers and ensemble learning algorithms for identifying immune checkpoint inhibitor (ICI) responders (AUC = 0.72) [20]. However, the application of these computational approaches to delineate RT‐specific immune determinants in cervical cancer remains underdeveloped. Current predictive models predominantly rely on radiomic features, lacking biological interpretability and actionable therapeutic targets [21, 22]. To address this critical gap, we propose an integrative machine learning paradigm that synergizes single‐cell transcriptomic resolution with bulk tissue profiling. This mechanistically informed predictive framework aims to identify radiation‐responsive biomarkers and facilitate precision RT decision‐making.

In this study, we systematically characterized RT‐induced remodeling of the tumor immune microenvironment (TIME), including macrophage polarization, enhanced T cell and NK cell cytotoxicity, and exacerbated T cell exhaustion. We further uncovered a critical role of the C3/C3AR1 axis in mediating epithelial cell‐macrophage interactions, which significantly dictated RT efficacy. Through multi‐modal integration and rigorous feature selection, we identified 8 core immune determinants from 25 radiation‐responsive biomarkers. These were integrated into the Cervical Cancer Radiotherapy Immune‐response Model (CCRTIM) using 15 machine learning algorithms, demonstrating high prognostic accuracy (AUC = 0.76). Our findings delineate the dynamic immune reorganization post‐radiation and establish clinically actionable biomarkers for prognosis prediction, offering new avenues for personalized treatment strategies.

## 2 Results

### 2.1 Single‐Cell Sequencing Reveals RT‐Driven Remodeling of the Cervical Cancer Tumor Microenvironment

**FIGURE 1:**
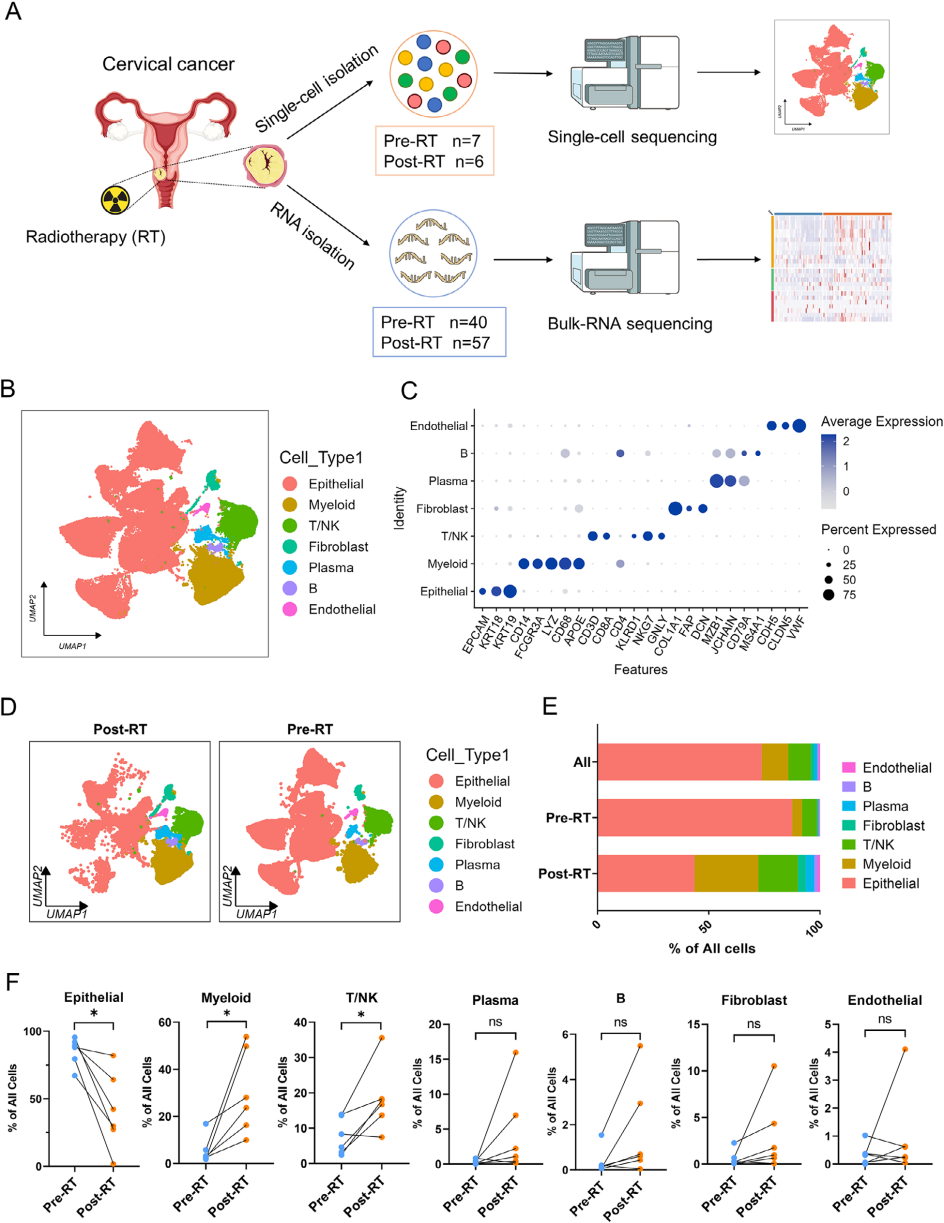
Single‐cell dissection of radiotherapy‐driven tumor microenvironment remodeling in cervical cancer. (A). Schematic of experimental design. Tumor biopsies from 7 cervical cancer patients collected pre‐radiotherapy (pre‐RT, *n* = 7) and post‐radiotherapy (post‐RT, *n* = 6) were subjected to scRNA‐seq, with validation bulk RNA‐seq cohorts (pre‐RT *n* = 40; post‐RT *n* = 57). (B). Integrated UMAP visualization of tumor microenvironment components, colored by seven major cell lineages: Epithelial, Myeloid, T/NK, (B), Plasma, Fibroblasts, and Endothelial. (C). Dot plot displaying lineage‐specific canonical marker expression levels (color intensity) and detection frequencies (dot size). (D). Comparative UMAP landscapes post‐RT vs pre‐RT. (E). Stacked bar plot showing proportional shifts in major lineages between pre‐RT and post‐RT. F. Paired analysis of cell proportion dynamics across individual patients (pre‐RT to post‐RT). ^*^ *p* <0.05, ns, non‐significant, *p* >0.05. Statistical analysis was performed using two‐tailed paired Student's *t*‐test (F).

To delineate RT‐induced remodeling of the cervical cancer tumor microenvironment (TME), we performed single‐cell RNA sequencing (scRNA‐seq) on 13 tumor specimens obtained from 7 patients (7 pre‐RT and 6 post‐RT samples). All tissue samples were histologically confirmed as tumors by pathological review, with representative hematoxylin and eosin (H&E) staining images provided (Figure S1A). Additionally, an In‐House cohort of 97 independent tumors bulk RNA sequencing (40 pre‐RT and 57 post‐RT) was conducted to validate the single‐cell findings (Figure 1A). Detailed clinical characteristics of the cohorts are provided in Tables 1, 2 and Table S1.

**TABLE 1:** Patient information of scRNA‐seq samples.

Patient ID	Age	HPV infection	Figo Stage	Treatment	Sample name	Radiotherapy	
1	75	HPV 16	IIIc1	CCRT	A‐1	Pre‐RT
					A‐2	Post‐RT
2	63	HPV 18	IIIa2	CCRT	CC‐1	Pre‐RT
					CC‐2	Post‐RT
3	56	HPV 16	IVa	CCRT	EE‐1	Pre‐RT
					EE‐2	Post‐RT
4	69	HPV 18	IVa	CCRT	GG‐1	Pre‐RT
					GG‐2	Post‐RT
5	78	HPV 58	IIIc2	CCRT	JJ‐1	Pre‐RT
					JJ‐2	Post‐RT
6	60	HPV 16	IIIc1	CCRT	YY‐1	Pre‐RT
					YY‐2	Post‐RT
7	58	NA	IIIb	NA^a^	XX‐1	Pre‐RT

CCRT: concurrent chemoradiotherapy

^a^ : The patient transferred to another hospital to receive treatment, and was lost of follow‐up.

**TABLE 2:** Clinical characteristics of patients who received bulk‐sequence.

Characteristic	
Age	median 60 (32–82)
Stage	
IIb	10 (15.6%)
III	43 (67.2%)
IVa	11 (17.2%)
Status	
dead	16 (25%)
alive	48 (75%)
Time to last follow‐up	
dead	median 469 days (243–1324)
alive	median 1472 days (673–2253)
Current chemoradiotherapy	56 (87.5%)

Following standard computational pipelines for single‐cell data processing, we performed quality control and clustering analysis on 110 047 high‐quality cells, which resolved into 23 transcriptionally distinct clusters (Figure S1B). These clusters were annotated as seven major cellular compartments based on canonical markers: 81 198 epithelial cells (Epithelial, marked by EPCAM, KRT18, and KRT19), 13 220 myeloid cells (Myeloid, marked by CD14, FCGR3A, LYZ, CD68, and APOE), 11 217 T and NK cells (T/NK, marked by CD3D, CD8A, CD4, KLRD1, NKG7, and GNLY), 688 B cells (B, marked by CD79A and MS4A1), 1503 plasma cells (Plasma, marked by MZB1 and JCHAIN), 1586 fibroblasts (Fibroblasts, marked by COL1A1, FAP, and DCN), and 635 endothelial cells (Endothelial, marked by CDH5, CLDN5, and VWF) (Figure 1B,C; Figure S1B,C). UMAP visualization and cellular frequency analysis further characterized the distribution of these cellular subsets across patients (Figure S1D,E).

Longitudinal analysis of cellular proportions revealed profound RT‐induced remodeling of the TME (Figure 1D–F). Epithelial cells exhibited a marked reduction from 87.46% pre‐RT to 43.61% post‐RT, while immune populations expanded significantly—myeloid cells increased from 4.46% to 28.69%, and T/NK cells from 6.76% to 17.77% (Figure 1E). Critically, paired analysis of individual patients (*n* = 6) confirmed at the single‐patient level the post‐RT decrease in epithelial cells and concurrent expansion of myeloid and T/NK cell populations (Figure 1F). These quantitative shifts demonstrate that RT drives a profound reconfiguration of the TME, characterized by a reduction in epithelial cells and an increase in immune cell populations.

### 2.2 RT Induced Epithelial Cell Complement Activation and Inflammatory Responses

**FIGURE 2:**
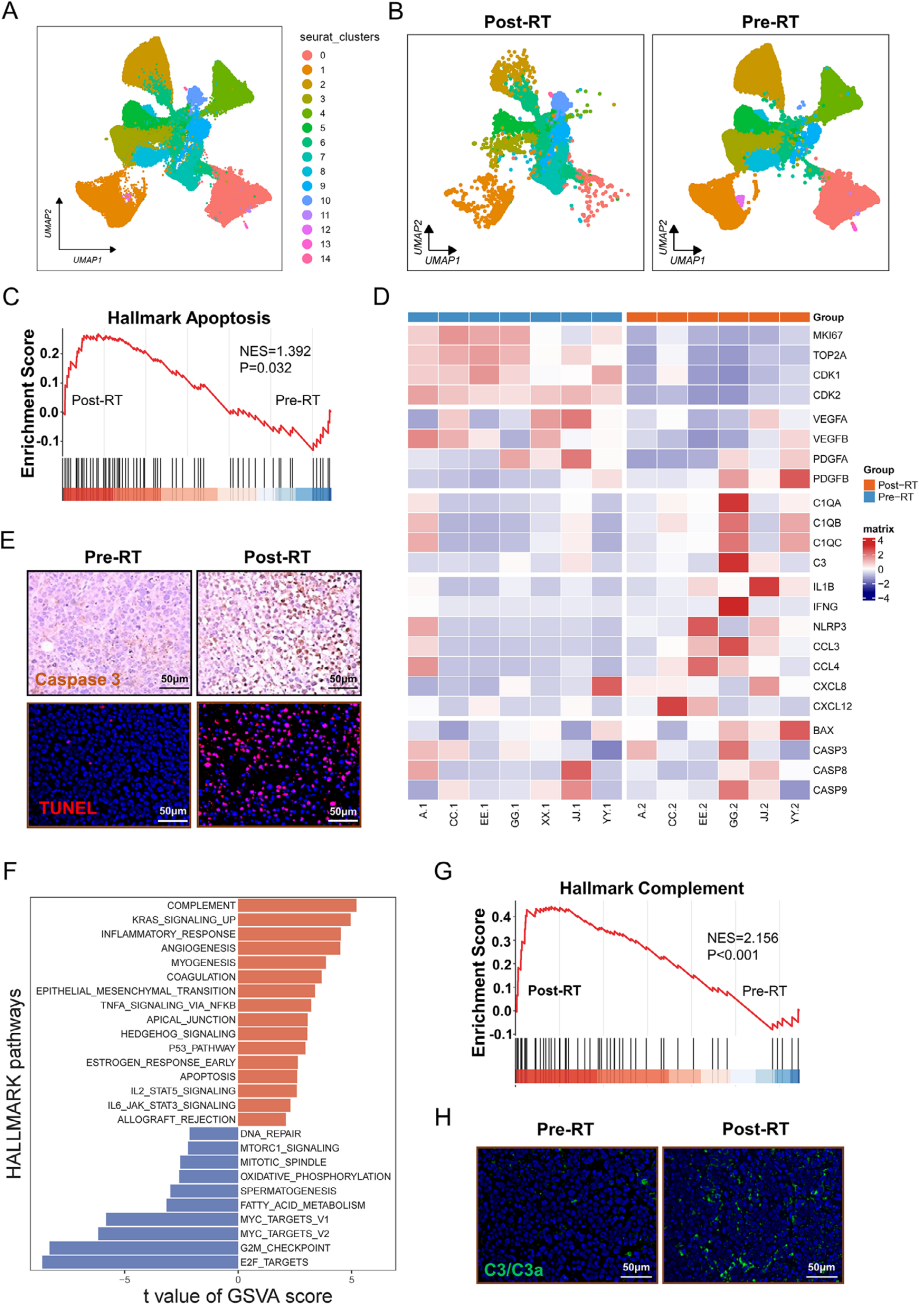
Radiotherapy induced epithelial cell complement activation and inflammatory responses. (A). UMAP projection of 15 transcriptionally distinct epithelial subclusters. (B). Comparative UMAP visualization of epithelial subclusters stratified by radiotherapy status (post‐RT vs pre‐RT). (C). Gene Set Enrichment Analysis (GSEA) demonstrating significant upregulation of apoptosis pathways in post‐RT epithelial cells. (D). Heatmap of functional gene signatures across treatment groups, including proliferation, growth factor, complement, inflammation and apoptosis modules. (E). Validation of epithelial apoptosis: Representative Caspase‐3 immunohistochemistry (IHC) and TUNEL staining in paired pre‐RT/post‐RT tumor sections. (F). Bar plot of Gene Set Variation Analysis (GSVA) scores for hallmark pathways in epithelial cells. (G). GSEA confirming significant enrichment of complement pathway in post‐RT epithelial cells. (H). Representative immunofluorescence (IF) staining of C3/C3a in paired pre‐RT/post‐RT tumor sections.

To further investigate the effects of RT on epithelial cells, we isolated epithelial cells and conducted a new round of clustering, identifying 15 distinct subpopulations based on marker genes (Figure 2A; Figure S2A). We observed a marked reduction in the number of epithelial cells following RT, despite their significant heterogeneity and dispersion across various clusters (Figure 2B; Figure S2B,C). To delineate the mechanisms underlying the reduction post‐RT, we performed Gene Set Enrichment Analysis (GSEA) on epithelial cells, which revealed marked enrichment of apoptosis pathway (Figure 2C). This was accompanied by upregulation of apoptosis‐related genes such as BAX, CASP3, and CASP9 (Figure 2D). Immunohistochemistry for CASP3 and TUNEL staining confirmed the increase in apoptosis after RT (Figure 2E; Figure S2D,E). Concurrently, cell cycle scoring indicated that post‐RT, epithelial cells were predominantly arrested in the G1 phase(Figure S2F,G), with downregulation of proliferation‐associated genes (Figure 2D). Additionally, the CNV (copy number variation) score of epithelial cells decreased after RT, suggesting that RT effectively eliminates cells with higher malignant potential (Figure S2H). Collectively, the enhanced apoptosis and proliferation arrest induced by RT account for the observed reduction in epithelial cell numbers.

To further explore the molecular changes in epithelial cells following RT, we performed differential gene expression analysis. Inflammatory response‐related genes, such as S100A7, S100A8, and CXCL6, were significantly upregulated, whereas cancer‐related genes, including STMN1, PTMA, and HMGB2, were markedly downregulated (Figure S2I). Gene Set Variation Analysis (GSVA) revealed that pathways associated with immune responses and inflammation, such as inflammation response and TNFA signaling via NFKB were significantly upregulated, while pathways related to cell proliferation, such as G2M checkpoint and DNA repair were downregulated (Figure 2F). Among these, the complement pathway ranked the highest enrichment score (Figure 2F), and GSEA analysis further confirmed the enrichment of the complement pathway (Figure 2G). Following RT, complement‐related genes, including C1QA, C1QB, and C3, were upregulated, and the increased C3/C3a protein and C3 mRNA levels in tumor tissues validated these findings (Figure 2D,H; Figure S2J,K). In summary, RT induces significant molecular and cellular changes in epithelial cells, including enhanced apoptosis, cell cycle arrest, and activation of inflammatory responses and complement.

### 2.3 RT Induced Macrophage M1 Polarization

**FIGURE 3:**
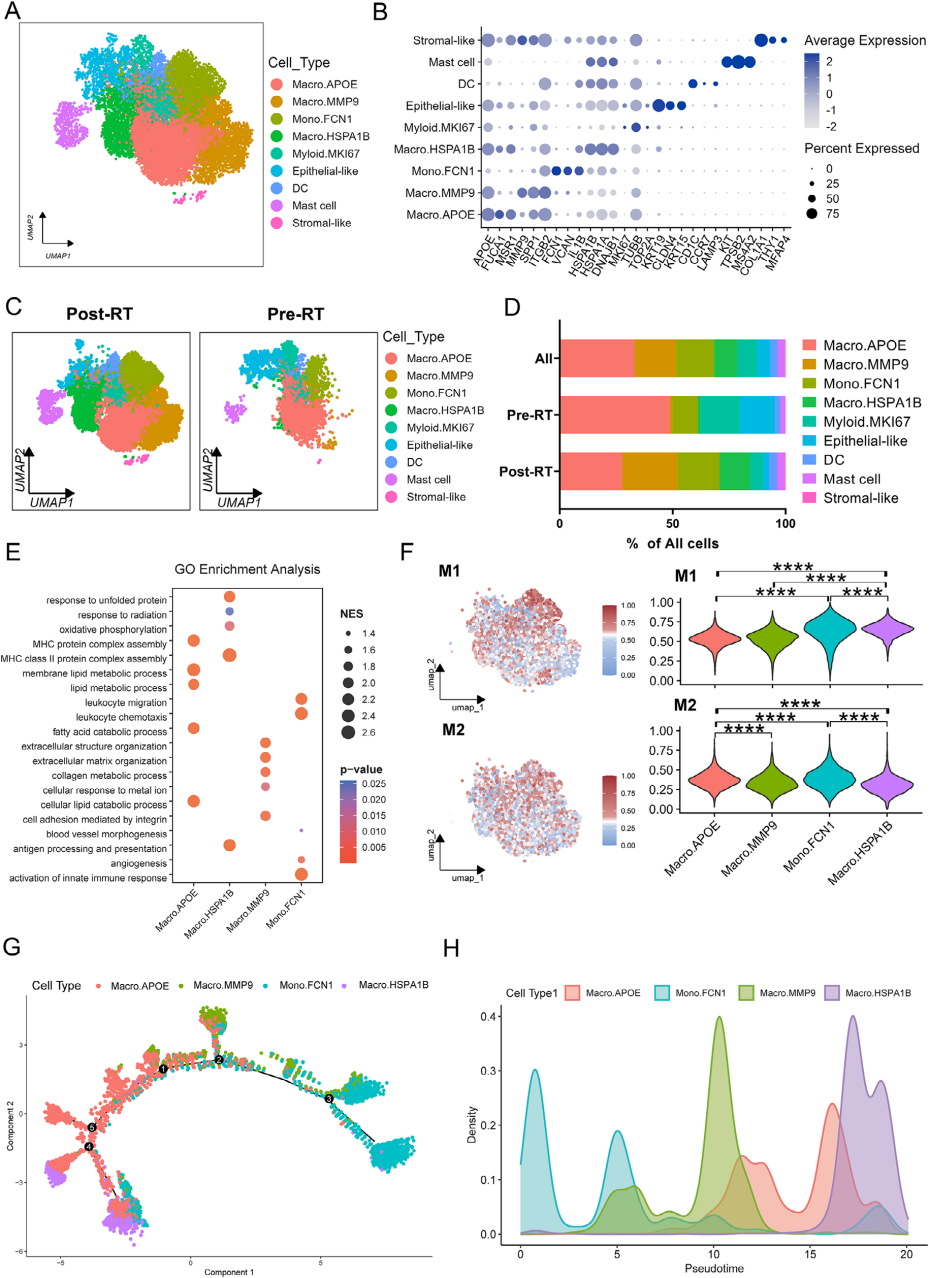
Radiotherapy induced the generation of radiation‐responsive M1‐like macrophage. (A). Integrated UMAP of 9 transcriptionally distinct myeloid subsets. (B). Dot plot of myeloid lineage‐defining markers with expression levels (color intensity) and detection frequencies (dot size). (C). UMAP visualization of myeloid composition stratified by radiotherapy status (post‐RT vs pre‐RT). (D). Stacked bar plot depicting proportional changes in myeloid cell populations between pre‐RT and post‐RT conditions. (E). Bar plot displaying normalized enrichment scores (NES) of GO‐enriched pathways in macrophage and monocyte clusters. (F). Feature plot (left) and violin plot (right) illustrating M1/M2 signature scores in macrophage and monocyte clusters. (G). Pseudotime trajectory of macrophages and monocytes. (H). The time–density plot illustrating the distribution of macrophage and monocyte subsets along the pseudotime trajectory. ^****^ *p* <0.0001. Statistical analysis was performed using the Wilcoxon rank‐sum test (F).

To investigate RT‐induced changes in immune cell dynamics, we first focused on myeloid cells due to their pronounced post‐RT expansion. Based on canonical cell markers and specific gene signatures, myeloid cells were classified into nine distinct subsets, including three macrophage subtypes (Macro.APOE, Macro.MMP9, and Macro.HSPA1B), a monocyte subtype (Mono.FCN1), a dendritic cell (DC) population, a mast cell population, a proliferative population (Myeloid.MKI67) and populations with epithelial or stromal features (Figure 3A,B; Figure S3A). Pre‐RT, Macro.APOE, Myeloid.MKI67, and the Epithelial‐like population were predominant, accounting for 48.7%, 17.5%, and 15.6% of the total myeloid population, respectively (Figure 3C,D). Post‐treatment, Macro.MMP9, Mono.FCN1, and Macro.HSPA1B showed marked increases, rising to 24.5%, 18.6%, and 13.4%, respectively (Figure 3C,D).

GSEA revealed that Macro.APOE was significantly enriched in lipid metabolism pathways, consistent with lipid‐associated macrophage characteristics [23, 24], while Macro.MMP9 was associated with extracellular matrix remodeling(Figure 3E) [25]. Mono.FCN1 exhibited inflammatory chemotactic properties and pro‐angiogenic capabilities, suggesting a dual role in inflammation and vascular remodeling (Figure 3E). Macro.HSPA1B expressed heat shock proteins genes, including HSPA1B, HSPA1A, and DNAJB1(Figure 3B), indicative of radiation response and oxidative stress features [26, 27], suggesting that this cell population may be specifically induced by RT, while also demonstrating strong antigen‐presenting capabilities (Figure 3E). Through analysis of macrophage‐related genes, we found that Macro.APOE exhibited high expression of M2 markers such as CD163 and MRC1, while Macro.HSPA1B showed elevated expression of M1 markers including CD86 and CD40, as well as HLA‐related genes (Figure S3B). Scoring the four subsets using established M1/M2 gene signatures validated these findings (Figure 3F).

Pseudotime trajectory analysis reconstructed the differentiation continuum from monocytes to macrophages, placing Mono.FCN1 monocytes at the origin, progressing through Macro.APOE (lipid‐associated) and Macro.MMP9 (matrix‐remodeling) intermediates, and culminating in Macro.HSPA1B macrophages with radiation‐induced stress response signatures (Figure 3G,H). Along the trajectory, M2‐associated genes (CD163 and MRC1) were highly expressed in intermediate stages (Macro.APOE and Macro.MMP9), consistent with immunosuppressive and tissue‐remodeling roles (Figure S3C,D). In contrast, M1‐associated genes (IL1B and TNF) were elevated in both initial (Mono.FCN1) and terminal (Macro.HSPA1B) stages, indicating a pro‐inflammatory phenotype (Figure S3C,D). Notably, MHC class II expression progressively increased, peaking in Macro.HSPA1B, reflecting their enhanced antigen‐presenting capacity (Figure S3C,D). Collectively, these findings suggest that RT‐induced macrophage expansion includes a continuum from immunosuppressive M2‐like subsets (Macro.APOE and Macro.MMP9) to M1‐like subsets (Macro.HSPA1B) with potent antigen‐presenting capabilities.

### 2.4 RT Enhances the Cytotoxicity of T Cells and NK Cells While Exacerbating Their Exhaustion

**FIGURE 4:**
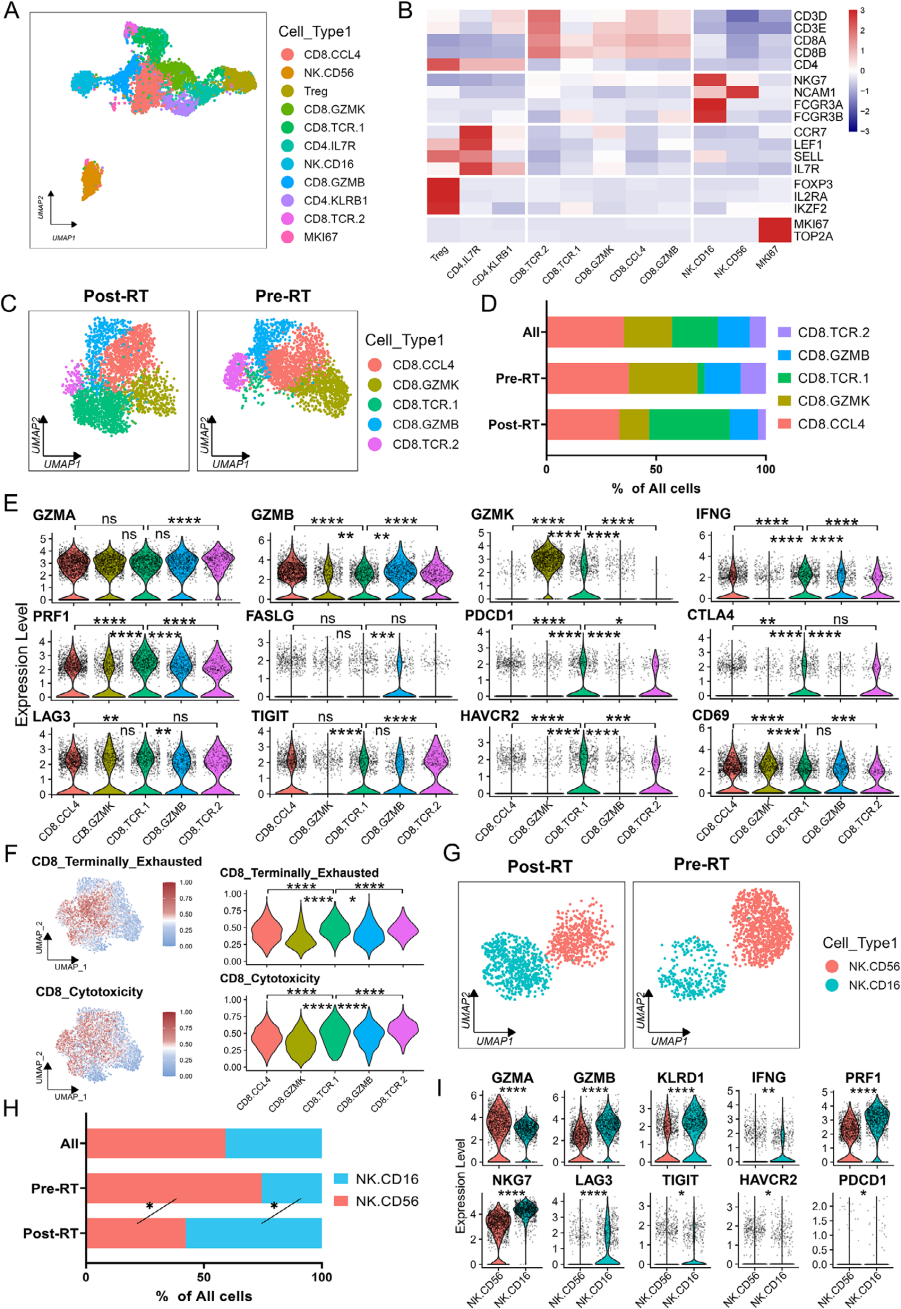
Radiotherapy enhances the cytotoxicity of CD8+ T cells and NK cells while exacerbating their exhaustion. (A). Integrated UMAP of 11 T and NK cell clusters. (B). Heatmap displaying the lineage‐specific canonical marker expression levels across the 11 T and NK cell clusters. (C). UMAP visualization of CD8+ T cell composition stratified by radiotherapy status (post‐RT vs pre‐RT). (D). Stacked bar plot depicting proportional changes in CD8+ T cell populations between pre‐RT and post‐RT conditions. (E). Violin plot showing expression of key T cell‐related genes in CD8+ T cell clusters. (F). Feature plot (left) and violin plot (right) illustrating exhaustion and cytotoxicity signature scores in CD8+ T cell clusters. (G). UMAP visualization of NK cell composition stratified by radiotherapy status (post‐RT vs pre‐RT). (H). Stacked bar plot depicting proportional changes in NK cell populations between pre‐RT and post‐RT conditions. (I). Violin plot showing expression of key T cell‐related genes in NK cell clusters. ^*^ *p* <0.05, ^**^ *p* <0.01, ^***^ *p* <0.001, ^****^ *p* <0.0001, ns, non‐significant, *p* >0.05. Statistical analysis was performed using the Wilcoxon rank‐sum test (E, F, H, and I).

T cells and NK cells were isolated based on canonical and highly variable marker genes and subsequently reclustered into 11 distinct subsets: 5 CD8+ T cells, 3 CD4+ T cells, 2 NK cells, and 1 proliferative subset (Figure 4A,B; Figure S4A). We first analyzed CD8+ T cell subsets, observing significant shifts in their proportions following RT. Pre‐RT, the CD8.CCL4, CD8.GZMK, and CD8.TCR.2 subsets predominated, whereas the CD8.TCR.1 subset expanded markedly post‐RT (Figure 4C,D; Figure S4B). The CD8.TCR.1 subset displayed elevated expression of cytotoxic markers (e.g., IFNG, PRF1, GZMB) alongside exhaustion‐associated genes (e.g., PDCD1, CTLA4, TIGIT), suggesting a dual phenotype of enhanced cytotoxicity coupled with exhaustion (Figure 4E). Cytotoxicity and exhaustion scores further validated this dichotomy [28, 29], confirming the CD8.TCR.1 subset as both highly cytotoxic and exhausted (Figure 4F). NK cell subsets also exhibited distinct changes post‐RT, with the CD56+ subset predominating before RT and the CD16+ subset expanding significantly afterward (Figure 4G,H; Figure S4C). Consistent with CD8+ T cells, the CD16+ NK subset upregulated cytotoxic markers (e.g., PRF1, GZMB) alongside exhaustion‐associated genes (e.g., LAG3, TIGIT), indicating a similar dual phenotype of enhanced cytotoxicity and exhaustion (Figure 4I).

Finally, we investigated CD4+ T cell subsets, which also increased in number post‐RT (Figure S4D–F). While the CD4.KLRB1 subset remained stable, the CD4.IL7R subset decreased, and Tregs expanded significantly (Figure S4F). The CD4.KLRB1 subset exhibited elevated expression of both cytotoxic (e.g., GZMA, GZMB, IFNG) and exhaustion markers (e.g., PDCD1, LAG3, CTLA4), whereas CD4.IL7R displayed a naive phenotype (Figure S4G). In summary, these results demonstrate that RT promoted the infiltration of T cells and NK cells and enhanced their cytotoxicity, while simultaneously exacerbating their exhaustion and driving a significant expansion of Tregs.

### 2.5 RT Enhanced Epithelial‐Macrophage Interaction via Complement Signaling

**FIGURE 5:**
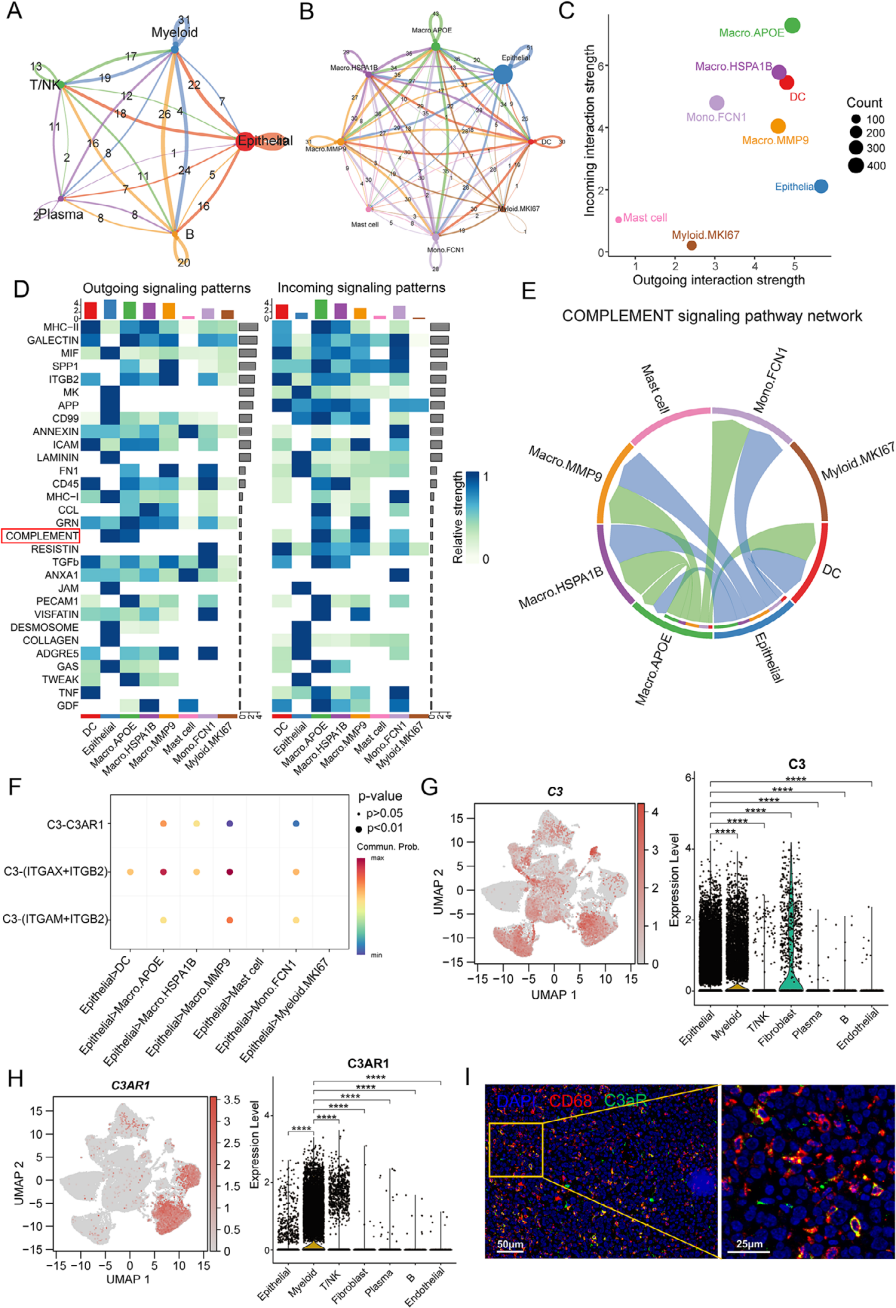
Radiotherapy enhances the crosstalk of epithelial and macrophage cells via complement pathway. (A). Chord diagram illustrating the interaction network between epithelial cells and immune cells post‐RT. (B). Chord diagram showing the interaction network between epithelial cells and myeloid subpopulations post‐RT. (C). Scatter plot depicting the outgoing and incoming interaction strength post‐RT. (D). Heatmap showing the top30 outgoing and incoming signaling pathways across different cell subpopulations post‐RT. (E). Interaction network of cell subpopulations mediated by the complement signaling pathway post‐RT. (F). Bubble plot displaying ligand‐receptor pairs involved in complement signaling. (G). Feature plot (left) and violin plot (right) illustrating the expression of C3 across different cell types. (H). Feature plot (left) and violin plot (right) illustrating the expression of C3AR1 across different cell types. (I). Representative immunofluorescence images showing colocalization of C3AR1 and CD68. ^****^ *p* <0.0001. Statistical analysis was performed using the Wilcoxon rank‐sum test (G and H).

To further explore the RT‐induced dynamics of the tumor microenvironment, we employed the CellChat package to analyze intercellular communication between epithelial and immune cells. Our analysis revealed that epithelial cells predominantly interact with myeloid cells, underscoring the significance of epithelial‐myeloid crosstalk in the microenvironment Post‐RT (Figure 5A). A detailed assessment revealed robust intercellular communication between epithelial cells and all myeloid subtypes (Figure 5B). Epithelial cells exhibited significantly enhanced outgoing signals, while macrophages (Macro.APOE, Macro.HSPA1B, Macro.MMP9), dendritic cells (DC), and monocytes (Mono.FCN1) displayed elevated incoming signals (Figure 5C), underscoring the dynamic bidirectional crosstalk between these cell populations following RT. A heatmap visualizing the major cell–cell communication pathways, which included complement signaling (Figure 5D). Building on our earlier findings of complement activation, we further characterized this pathway using chord diagrams (Figure 5E) and network centrality analysis (Figure S5A). These data confirmed that epithelial cells act as the principal source of complement signals targeting macrophages, monocytes, and dendritic cells. Ligand‐receptor analysis identified communication mediated by complement component C3 (C3) and its active fragment C3a's receptor C3AR1, as well as integrin complexes (ITGAX + ITGB2) and (ITGAM + ITGB2) (Figure 5F). C3, serving as the central effector molecule of the complement system, orchestrates myeloid cell reprogramming through the binding of its proteolytic fragment C3a to the receptor C3AR1 [30, 31, 32]. To delineate the functional crosstalk of the C3‐C3AR1 axis, we revealed that C3 was specifically enriched in epithelial cells, while C3AR1 was predominantly expressed in myeloid cells (Figure 5G,H). Immunofluorescence staining confirmed C3AR1 colocalization with CD68, indicating specific expression on macrophages (Figure 5I). Furthermore, analysis of bulk RNA sequencing data (In‐House cohort) confirmed significant enrichment of complement activation pathways, particularly the classical pathway, alongside upregulated expression of complement‐related genes following RT (Figure S5B,C). Collectively, these findings demonstrated that RT enhanced epithelial‐macrophage interactions via complement signaling, with epithelial cells acting as primary C3 senders and macrophages as major C3AR1 receivers.

### 2.6 The Complement C3‐C3AR1 Pathway Plays a Crucial Role in the Control of Cervical Cancer During RT

**FIGURE 6:**
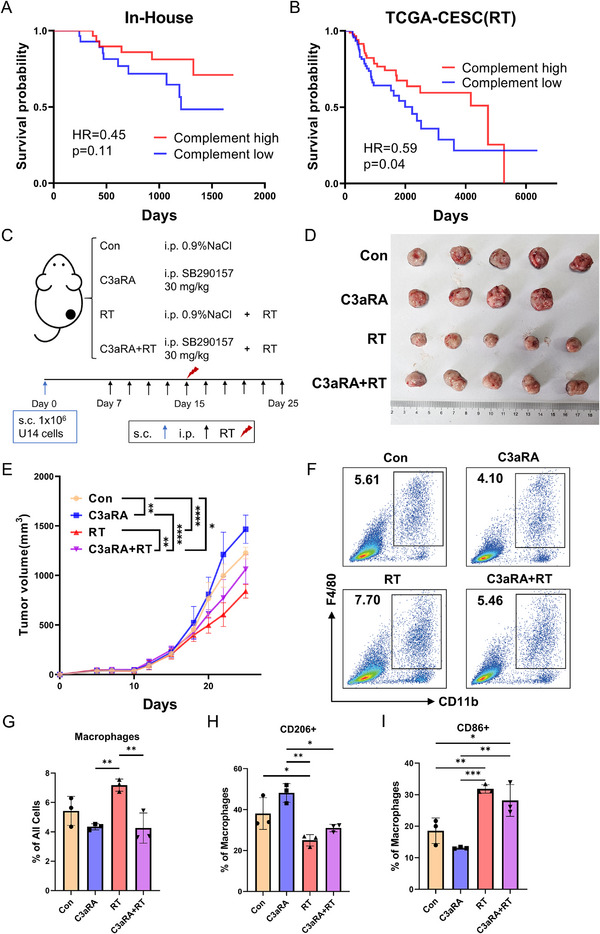
Blocking the complement C3a receptor impairs the efficacy of radiotherapy in tumor control. (A). Kaplan–Meier plot showing overall survival stratified by Complement score from In‐House cohort (*n* = 57) following RT. (B). Kaplan–Meier plot showing overall survival stratified by Complement score from TCGA‐CESC(RT) cohort (*n* = 187) following RT. (C). Schematic diagram of treatment and timeline for the mice radiotherapy experiment. (D). Representative images of tumors in every group collected on day 25. (E). Tumor growth curves of every group from day 0 to day 25. *n* = 5 per group. (F). Representative flow cytometry dot plots of CD11b+ F4/80+ macrophages’ frequencies in mice tumor. (G). Proportion of tumor‐infiltrating macrophages under different treatment conditions. (H). Proportion of CD206+ macrophages under different treatment conditions. (I). Proportion of CD86+ macrophages under different treatment conditions. Data presented as mean ± SEM. ^*^ *p* <0.05, ^**^ *p* <0.01, ^***^ *p* <0.001, ^****^ *p* <0.0001. Statistical analysis was performed using log‐rank (Mantel–Cox) test (A and B), two‐way ANOVA with Tukey's test (E), and one‐way ANOVA with Tukey's test (G, H, and I).

To investigate the role of the complement pathway in the RT response, we performed Kaplan‐Meier survival analysis using a composite Complement pathway score (derived via GSVA) in both our In‐House cohort and the TCGA‐CESC (RT) cohort. The results demonstrate that a higher complement score was significantly associated with prolonged survival in the TCGA cohort (*p* = 0.04) and showed a strong, consistent trend in our cohort (Figure 6A,B). Analysis of C3 gene expression alone revealed a similar trend (Figure S6A,B). Collectively, these findings support a positive association between complement pathway activation and a favorable prognosis following radiotherapy. To validate these findings, we established a murine cervical cancer model using U14 subcutaneous tumors and administered the C3AR1 antagonist SB290157 [33, 34] to block complement signaling. Following tumor irradiation, longitudinal tumor growth was monitored until endpoint analysis. The specific timeline and administration details are depicted in Figure 6C. C3AR1 antagonist markedly attenuated the tumor‐suppressive efficacy of RT (Figure 6D,E; Figure S6C). Flow cytometry analysis revealed that C3AR1 blockade significantly reduced macrophage infiltration post‐RT (Figure 6F,G). Further analysis revealed that blocking C3AR1 altered macrophage polarization, resulting in a higher proportion of CD206+ macrophages (M2‐like) and a reduction in CD86+ macrophages (M1‐like) (Figure 6H,I; Figure S6D,E). Our results establish that RT‐induced complement activation through the C3‐C3AR1 axis enhances anti‐tumor immunity by promoting macrophage infiltration and polarization.

### 2.7 A CCRTIM Model Predicts RT Prognosis in Cervical Cancer

**FIGURE 7:**
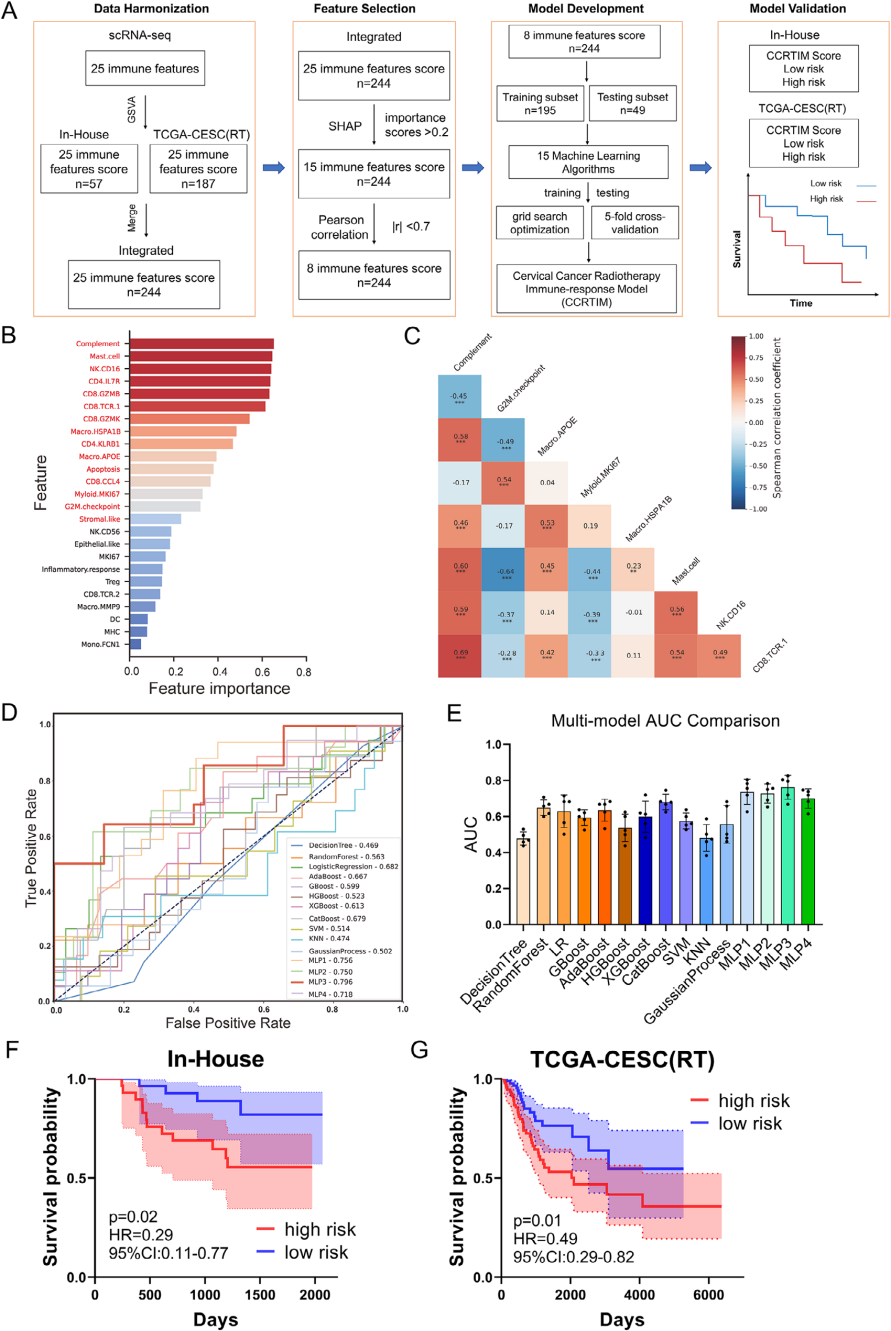
Developing Cervical cancer radiotherapy‐immune response model (CCRTIM) model integrated scRNA‐seq and bulk‐seq predicts radiotherapy outcomes in cervical cancer. (A). Schematic of the RTIM model development workflow. Single‐cell RNA‐seq‐derived immune features (25 immune‐related signatures) were integrated with bulk RNA‐seq data from In‐House cohort (*n* = 57) and TCGA‐CESC(RT) cohort (*n* = 187). (B). SHapley Additive exPlanations (SHAP) analysis ranked feature importance, identifying 15 key predictors (importance score >0.2). (C). Pearson correlation heatmap refined the feature set to eight non‐redundant predictors, including Complement, G2M.checkpoint, Macro.APOE, Myeloid.MKI67, Macro.HSPA1B, Mast cell, NK.CD16, and CD8.TCR.1. (D). Receiver Operating Characteristic (ROC) curves of top‐performing machine learning models, with Multilayer Perceptron (MLP3) achieving the highest AUC (0.762 ± 0.004). (E). Bar plot comparing model performance (AUC) across 15 algorithms, highlighting MLP3 superiority. (F). Kaplan–Meier plot showing overall survival stratified by CCRTIM score from In‐House cohort (*n* = 57). (G). Kaplan–Meier plot showing overall survival stratified by CCRTIM score from TCGA‐CESC (RT) cohort (*n* = 187). Statistical analysis was performed using log‐rank (Mantel–Cox) test (F and G).

To investigate the relationship between RT‐induced immune dynamics and clinical outcomes, we developed a Cervical Cancer Radiotherapy Immune‐response Model (CCRTIM) (Figure 7A). First, we mapped 25 immune features derived from single‐cell sequencing data to two independent cohorts—the In‐House cohort (*n* = 57) and TCGA‐CESC (RT) cohort (*n* = 187)—using the Gene Set Variation Analysis (GSVA) algorithm. Second, we evaluated data consistency between cohorts through principal component analysis (PCA) and uniform manifold approximation and projection (UMAP) (Figure S7A,B), confirmed that samples from both cohorts share highly similar feature profiles and lack distinct cluster structures, and merged them into an Integrated‐cohort (*n* = 244) to enhance model robustness. In addition, given that our study cohort encompassed both RT and concurrent chemoradiotherapy (CCRT, RT with weekly cisplatin) regimens, we rigorously evaluated data consistency across the treatment modalities. No separation was observed between the two groups (Figure S7C). All data were randomly split into training (80%, *n* = 195) and validation (20%, *n* = 49) subsets. Subsequently, a two‐tiered feature selection strategy was employed: SHapley Additive exPlanations (SHAP) analysis prioritized 15 features with importance scores >0.2 (Figure 7B); Pearson correlation filtering (|r| >0.7) eliminated redundant features, yielding 8 non‐redundant predictors including Complement, G2M.checkpoint, Macro.APOE, Myeloid.MKI67, Macro.HSPA1B, Mast cell, NK.CD16, and CD8.TCR.1 (Figure 7C; Figure S7D). Next, we systematically constructed and evaluated prognostic models using 15 machine learning architectures. For each model, grid search optimization (100 iterations/algorithm) and 5‐fold cross‐validation were performed to ensure unbiased performance. The optimal model identified was an 8‐feature three‐layer Multilayer Perceptron (MLP3) with ReLU activation, achieving an AUC of 0.762 ± 0.004 (Figure 7D,E and Table 3). This model, designated CCRTIM, was finalized for prognostic prediction. Finally, CCRTIM was validated across both cohorts. Risk stratification based on CCRTIM scores revealed significant survival disparities between high‐ and low‐risk groups. The low‐risk group (lower 50%) exhibited superior overall survival in the In‐House cohort (HR = 0.29, *p* = 0.02; Figure 7F) and TCGA‐CESC (RT) cohort (HR = 0.49, *p* = 0.01; Figure 7G). In conclusion, the CCRTIM model accurately predicts radiotherapy outcomes in cervical cancer by decoding immune dynamics, providing a clinically actionable tool for risk stratification.

**TABLE 3:** Multi‐model AUC Comparison.

Methods	AUC	Methods	AUC	Methods	AUC
DecisionTree	0.478 ± 0.001	HGBoost	0.537 ± 0.005	GP	0.557 ± 0.009
RandomForest	0.649 ± 0.001	XGBoost	0.599 ± 0.006	MLP1	0.737 ± 0.004
LR	0.629 ± 0.006	CatBoost	0.680 ± 0.002	MLP2	0.727 ± 0.002
GBoost	0.593 ± 0.002	SVM	0.575 ± 0.002	MLP3	0.762 ± 0.004
AdaBoost	0.635 ± 0.003	KNN	0.481 ± 0.004	MLP4	0.700 ± 0.002

## 3 Discussion

RT exerts profound yet paradoxical effects on the tumor immune microenvironment, presenting both therapeutic opportunities and resistance challenges [35, 36]. Through systematic integration of longitudinal single‐cell transcriptomics (*n* = 13 specimens) and bulk RNA sequencing (*n* = 97 tumors) in cervical cancer patients, we systematically resolved the spatiotemporal dynamics of radiation‐induced immune remodeling. Notably, our findings align with emerging evidence on innate immune activation in RT responses [16], particularly through complement cascade initiation (C3 upregulation) and macrophage polarization toward an antigen‐presenting M1‐like phenotype (Macro.HSPA1B). Crucially, we identified the coexistence of enhanced cytotoxic potential (GZMB/PRF1 upregulation) and progressive exhaustion (PDCD1/TIGIT elevation) in T/NK cell populations, revealing an inherent dichotomy in RT's immunomodulatory effects. By synthesizing these multidimensional immune features, we developed a biologically interpretable Cervical Cancer Radiotherapy Immune‐response Model (CCRTIM) that translated single‐cell insights into clinical‐grade prognostic predictions (AUC = 0.76), establishing a mechanistically informed framework for personalizing RT strategies.

The complement system, a cornerstone of innate immunity, plays pivotal roles in antimicrobial defense, inflammatory regulation, and humoral immunity [37, 38]. Our study unveils a previously underappreciated role of radiation‐induced complement signaling in shaping the cervical cancer immune microenvironment. We demonstrate that RT triggers epithelial stress responses characterized by dual activation of apoptotic machinery (BAX/CASP3) and complement cascade (C1Q/C3 upregulation), with single‐cell resolution revealing epithelial cells as the predominant source of C3 secretion. Through ligand‐receptor topology analysis (CellChat), we mechanistically delineated C3/C3AR1‐mediated epithelial‐macrophage crosstalk, revealing spatial coordination between complement‐producing epithelial niches and C3AR1+ macrophages. While tumor‐derived C3 promotes ovarian cancer invasion by enhancing epithelial‐mesenchymal transition (EMT) [39], and suppresses CD8+ T cell cytotoxicity to accelerate hepatocarcinoma progression [40], our work establishes a radiation‐specific paradigm in cervical cancer where C3/C3AR1 signaling correlates positively with RT efficacy. This tissue‐specific complement rewiring stands in stark contrast to its tumor‐promoting roles in other malignancies, as evidenced by in vivo validation: C3AR1 antagonism (SB290157) attenuated RT‐mediated tumor control in murine models, concurrent with M1‐like macrophage reduction and T cell exhaustion exacerbation. These findings align with Surace et al.’s demonstration of compromised RT efficacy upon C3/C5 ablation in melanoma models [41], yet extend beyond by elucidating the epithelial‐immune coordination mechanism. Collectively, we position the C3/C3AR1 axis as a master regulator of RT response through spatiotemporal immune remodeling, establishing targeted complement modulation as a novel radiosensitization strategy.

Tumor‐associated macrophages (TAMs) are pivotal components of the tumor microenvironment, typically exerting immunosuppressive functions to promote tumor progression [42, 43]. In cervical cancer, hypoxia‐driven CCL8 secretion recruits TAMs, which further facilitate lymphangiogenesis and angiogenesis to support metastasis [44, 45, 46]. Emerging evidence reveals that RT induces dual functional reprogramming of tumor‐associated macrophages, characterized by the simultaneous upregulation of immunostimulatory markers (e.g., HMGB1 and ISG15) and immunosuppressive markers (e.g., SIRPA and IDO1), accompanied by a significant expansion of macrophage populations within the irradiated tumor microenvironment [47]. A distinct macrophage subset emerged with oxidative stress adaptation and enhanced antigen‐presenting capacity (Macro.HSPA1B), displaying M1‐like characteristics. This functional plasticity appears critical, as macrophage depletion (via clodronate liposomes) reduced RT efficacy in breast (4T1) cancer models [48]. Contrastingly, studies in colorectal (MC38) and pancreatic (KPC) cancer models demonstrated improved tumor control with CSF1R blockade to eliminate macrophages [49]. These conflicting outcomes may arise from tissue‐specific TAM heterogeneity or patient variability. For instance, cervical cancer macrophages under RT may adopt unique functional states distinct from gastrointestinal TAMs. Nevertheless, targeting macrophage‐RT crosstalk remains a promising therapeutic strategy to optimize combination therapies.

Our study delineates paradoxical dynamics in T and NK cell populations within the irradiated cervical cancer microenvironment. RT significantly increased CD8+ T cell and CD56+ NK cell proportions, accompanied by elevated effector molecules (GZMB, IFNG, PRF1), indicating enhanced anti‐tumor potential. Conversely, we observed concurrent expansion of immunosuppressive elements: regulatory T cells (Tregs) increased with CD8+ T cell proportions upregulated exhaustion markers (PDCD1, LAG3, CTLA4). This duality mirrors observations in lung cancer RT, where early cytotoxic activation precedes exhaustion‐mediated resistance [50]. These temporal shifts in immune functionality—activation preceding suppression—suggest an optimal therapeutic window for combination strategies. For instance, PD‐1/CTLA‐4 blockade administered during the cytotoxic phase may sustain anti‐tumor effects while counteracting exhaustion [51, 52].

Building upon radiation‐induced immune microenvironment remodeling, we developed the Cervical Cancer Radiotherapy Immune‐response Model (CCRTIM) to predict cervical cancer outcomes with high accuracy (AUC = 0.762 ± 0.004). CCRTIM integrates mechanistically relevant biomarkers, including complement pathway activity, Macro.HSPA1B macrophage, and CD8.TCR.1 T cell signatures. These biomarkers were functionally validated through C3AR1 blockade experiments, directly linking model parameters to biological efficacy. Intriguingly, mast cells were retained as a feature with high SHAP weight in the final model, underscoring their potential functional importance in the context of radiotherapy. Biologically, mast cells act as a primary source of histamine. In concert with complement activation, histamine may promote an anti‐tumor microenvironment by driving M1‐like macrophage polarization, as characterized by the elevated expression of OSM, IL‐6, IL‐12, and TNF‐α [53]. Furthermore, mast cell‐derived histamine has been shown to enhance IL‐16‐mediated CXCR3 expression in tumor‐associated macrophages, thereby potentiating the efficacy of immune checkpoint blockade [54]. Thus, within the specific context of radiotherapy‐induced complement activation, mast cells likely function as critical catalysts that amplify immune remodeling toward an anti‐tumor phenotype. While this mechanism provides biological plausibility for the predictive power of CCRTIM, further experimental investigation is warranted to fully validate this hypothesis.

This biologically interpretable framework addresses a critical gap in radiation oncology. Conventional radiomics models, which typically rely on CT or MRI features, can achieve high predictive performance (e.g., an AUC of 0.86 was reported for an MRI‐based model) [55], yet they often lack biological interpretability and do not yield readily actionable therapeutic targets. Similarly, while classifiers derived from PET/CT radiomics correlate with tumor morphology [56], they provide limited insight into the underlying mechanisms of treatment resistance. Conversely, CCRTIM's single‐cell‐derived features elucidate functional pathways governing radio‐response, such as macrophage‐mediated antigen presentation and T cell exhaustion dynamics. By bridging multidimensional immune profiling with clinical outcomes, CCRTIM establishes a new paradigm for precision RT, where predictive models directly inform therapeutic strategies.

In conclusion, our study establishes RT as a pivotal modulator of epithelial‐macrophage crosstalk within the cervical cancer immune microenvironment, mechanistically governed by the complement C3/C3AR1 axis. The CCRTIM model surpasses conventional imaging‐based prognostic tools by integrating biologically grounded immune signatures—such as complement activation and macrophage populations—with radiation responsiveness, achieving improved predictive accuracy and interpretability. By prioritizing these mechanistic biomarkers over opaque radiomic features, this framework provides a clinically actionable pathway to enhance therapeutic efficacy through precision RT regimens tailored to individual immune dynamics.

## 4 Experimental Section

### 4.1 Human Specimens

Between Jan 17, 2022 and Aug 4, 2022, we collected fresh cervical cancer tissue samples from patients with locally advanced cervical cancer (LACC), by biopsy before (Pre‐RT) and at the 11th faction of external beam RT (Post‐RT). Six patients underwent radical RT, and one patient transferred to another hospital to receive treatment. The treatment consisted of a 45 Gy external beam RT given at 5 fractions per week, followed by image‐guided brachytherapy which consisted of 30–35 Gy in 5–6 fractions; all of them received concurrent chemotherapy. Our SC‐cohort was consisted of 13 samples, including 12 matched pre‐ and post‐RT tumor tissues from 6 patients, and 1 pre‐RT tissue. The characteristics for individual patients are given in Table 1.

A bulk RNA‐seq cohort (In‐House cohort) was used to validate the single‐cell findings and develop the Cervical Cancer Radiotherapy Immune‐response Model (CCRTIM). It was consisted of 97 samples collected before or 2 weeks from initiation of RT from 64 patients, including 66 paired pre‐ and post‐RT tumor tissues from 33 patients, 24 post‐RT, and 7 pre‐RT samples. All of these patients received radical RT, and concurrent chemotherapy was given to 56 of them. 16 patients died during the follow‐up, with a median overall survival of 469 (243‐1324) days; and the rest 48 patients were alive at the last follow‐up, with a median follow‐up time of 1472 (673‐2253) days. The characteristics for individual patients are given in Table 2 and supplementary Table 1. The research was conducted with approval from the Ethics Committee of Renji Hospital, Shanghai Jiao Tong University School of Medicine (No. LY2024‐288‐C) and all the patients provided written consent for the use of their tissue samples in the study.

### 4.2 Mice

6–8 weeks old male C57BL/6 J mice were purchased from Shanghai JieSiJie Laboratory Animals Co., LTD (Shanghai, China) for U14 cervical carcinoma model establishment. Animals were housed under controlled conditions: 12/12‐h light/dark cycle, 22 ± 1°C ambient temperature, 50 ± 10% humidity, with ad libitum access to standard chow and filtered water. For subcutaneous tumor induction, 1 × 10^6^ U14 cells suspended in 100 µL PBS were unilaterally inoculated into the right dorsal flank. Tumor dimensions were measured every two‐ or three‐days after tumor formation using digital calipers, with volume calculated as V = (W^2^ × L)/2, W = tumor width, L = tumor length, V = tumor volume. All animal experiments were conducted following a protocol approved by the Institutional Animal Care and Use Committee (IACUC) of Shanghai Jiao Tong University (No. 2025007).

### 4.3 Radiotherapy for Mice

When tumor volume reached an average of 200 mm^3^, mice were anesthetized and immobilized for localized radiotherapy. Tumors were irradiated with a single dose of 12 Gy using a medical linear accelerator (Elekta Synergy) with 6 MV X‐rays at a dose rate of 600 MU/min. The rest of the body was shielded to minimize radiation exposure to normal tissues.

### 4.4 C3AR1 Antagonist Treatment for Mice

Tumor‐bearing mice were randomly assigned to four groups: Con, C3aRA, RT, and C3aRA + RT. Starting on day 7 post‐inoculation, mice in the C3aRA and C3aRA + RT groups received intraperitoneal (i.p.) injections of SB290157 (30 mg/kg in saline; MedChemExpress, HY‐101502A) every other day, while control groups received equivalent volumes of saline. Localized radiotherapy was administered to the RT and C3aRA + RT groups on day 15. Treatment continued until day 25 (10 total administrations). Tumor volumes were monitored throughout, and tumors were harvested and weighed at the endpoint.

### 4.5 Cell Lines

The murine cervical carcinoma cell line U14 (RRID:CVCL_9U56) was purchased from Cellverse Co., Ltd. (Shanghai, China). Cell line authentication was performed by short tandem repeat (STR) profiling, and routine mycoplasma testing confirmed the absence of contamination. Cells were cultured in high‐glucose DMEM (Gibco, USA) supplemented with 10% heat‐inactivated 10% fetal bovine serum and 1% penicillin‐streptomycin. Cultures were maintained at 37°C in a humidified 5% CO_2_ atmosphere with medium replacement every 48 h, and passaged at 80%–90% confluence using 0.25% trypsin‐EDTA.

### 4.6 Sample Preparation and Single‐Cell Isolation

Fresh cervical carcinoma specimens were enzymatically dissociated in sterile conditions. Tissue fragments were washed twice with cold RPMI 1640 (Gibco) containing 0.04% BSA, then minced into 0.5 mm^3^ pieces. Digestion was performed in 5 mL of collagenase IV (2 mg/mL, Gibco) and DNase I (1 mg/mL, Invitrogen) at 37°C for 60 min with gentle agitation every 5 min. The suspension was filtered through 40 µm strainers and centrifuged (300g, 5 min, 4°C). Erythrocytes were lysed using Pharm Lyse (BD; 1:1 v/v, 10 min, 4°C). After washing twice in basal medium, cells were resuspended in PBS for quantification and viability assessment (≥85% viability) using a Luna cell counter with trypan blue exclusion.

### 4.7 Single‐Cell Library Construction and Sequencing

The freshly prepared single‐cell suspension was adjusted to 700–1200 cells/µL according to the 10×Genomics Chromium Next GEM Single Cell 3ʹ Reagent Kits v3.1 (No. 1000268) Operation manual for computer and library construction. The constructed library was sequenced using the Illumina Nova 6000 PE150 platform.

### 4.8 scRNA‐Seq Data Processing

Single‐cell RNA sequencing data were processed with Cell Ranger (v6.1.2) using the GRCh38.p13 genome. Raw UMI matrices underwent quality control in Seurat v5.2.1, excluding cells with <500 or >6000 genes, >10% mitochondrial genes, or >1% hemoglobin (HBA1/HBA2/HBB) expression. Genes detected in <200 cells were removed. Doublets were identified by DoubletFinder [57] v2.0.3 (pK = 0.09, 5% expected rate). Data were normalized via LogNormalize (scale factor = 10 000) and batch‐corrected using Harmony [58] v1.2.3 (theta = 2, lambda = 1) on top 30 principal components.

### 4.9 Single‐Cell RNA‐Seq Data Clustering and Annotation

The normalized data were used to identify gene features with high cell‐to‐cell variations by utilizing the FindVariableFeatures function. The top 2000 highly variable genes were used to scale the data by using the ScaleData function. Principal‐component analysis (PCA) was performed to reduce the dimensionality with RunPCA function. The FindNeighbors and FindClusters functions were consecutively used to perform a graph‐based clustering and find the optimal cluster resolution. Cells were visualized using a 2D Uniform Manifold Approximation and Projection (UMAP) algorithm with the RunUMAP function. The FindAllMarkers function was used to identify marker genes of each cluster. In the first‐round clustering, epithelial cells (Epithelial), myeloid cells (Myeloid), T/natural killer cells (T/NK), B cells, plasma B cells (Plasma), fibroblasts (Fibroblasts), and endothelial cells (Endothelial) were identified using unsupervised clustering, following the strategy described above. These cell types were characterized by their high expression levels of EPCAM/KRT18/KRT19, CD14/FCGR3A/CD68, CD3D/CD8A/NKG7, CD79A/MS4A1, MZB1/JCHAIN, COL1A1/FAP/DCN, and CDH5/CLDN5/VWF, respectively. For refined characterization, Epithelial, Myeloid, and T/NK compartments underwent secondary clustering, revealing functional subsets according to their specific markers.

### 4.10 Bulk‐RNA Sequencing

Tumor specimens were flash‐frozen in liquid nitrogen and stored at −80°C. RNA isolation was performed using TRIzol Reagent (Invitrogen, USA) following mechanical homogenization. RNA quality was assessed via Qubit 4.0 Fluorometer (Thermo Fisher Scientific, USA) and Bioanalyzer 2100 (Agilent Technologies, USA). Strand‐specific libraries were prepared with the NEBNext Ultra II RNA Library Prep Kit (NEB) through poly‐A selection and fragmentation (250–300 bp). Sequencing was conducted on an Illumina NovaSeq 6000 system (S4 flow cells) in 150 bp paired‐end mode, yielding ∼40 million reads per sample.

### 4.11 Gene Set Enrichment Analysis (GSEA)

Differentially expressed genes (DEGs) post‐RT were identified using the FindMarkers function (Seurat v5.2.1) for single‐cell RNA‐seq data and the DEG2 package (v1.42.1) for bulk RNA‐seq data. Upregulated genes from both datasets were used for functional annotation, respectively. Enrichment of hallmark pathways (MSigDB HALLMARK v7.5.1) and KEGG pathways (KEGG v2023.2) was assessed using clusterProfiler (v4.10.1). Specific pathways were visualized via ggplot2 (v3.5.1).

### 4.12 Gene Set Variation Analysis (GSVA)

Hallmark pathway (MSigDB v7.5.1) activity scores post‐RT were computed via GSVA (v1.50.5). Significant pathways were defined by |log2 fold change| >0 and adjusted *p* <0.05. Results were visualized through directional bar plots (ggplot2 v3.5.1), highlighting upregulated (firebrick) and downregulated (navy) pathways. Pathway labels were dynamically positioned to avoid overlap, with axes scaled to maximize discriminability of t‐statistics.

### 4.13 Calculation of M1/M2 and Exhaustion/Cytotoxicity Scores

Macrophage polarization states and CD8+ T‐cell functional profiles were calculated using the AddModuleScore function in Seurat (v5.2.1), using established gene signatures. Scores were projected onto UMAP embeddings to visualize spatial distributions across subclusters. Violin plots were generated using ggplot2 (v3.5.1) to compare score distributions between annotated cell subsets.

### 4.14 Cell–Cell Crosstalk Analysis

Interactions between epithelial cells and immune/myeloid cells were analyzed using the CellChat (v1.6.1) package [59]. We quantified the number and interaction strength of outgoing and incoming signaling pathways between these cell populations. For epithelial‐myeloid interactions, we further focused on the complement signaling network and identified specific ligand‐receptor pairs contributing to this pathway.

### 4.15 Pseudotime Trajectory Analysis

Pseudotime ordering of macrophage/monocyte differentiation trajectories was performed using monocle (v2.32.0) following established analytical workflows [60, 61]. Processed integrated scRNA‐seq transcriptomic matrices were subjected to unsupervised trajectory inference, where pseudotime ordering was conducted through unsupervised clustering of differentially expressed genes (DEGs) across four developmentally related myeloid subsets (adjusted *p*<0.01). Dimension reduction was implemented via graph‐based dimensionality reduction (DDRTree algorithm) with elastic principal graph construction. Cell state transitions along the differentiation continuum were computationally reconstructed using default parameters for branch detection and pseudotime calculation.

### 4.16 Flow Cytometry Analysis

Tumors were dissociated into single‐cell suspensions using the enzymatic protocol established for clinical specimens. Cells were resuspended in PBS (1 × 10^6^ cells/mL) containing 2% FBS for staining. Viability discrimination was performed with Zombie Aqua Fixable Viability Kit (BioLegend, 423101; 1:200) for 15 min at 4°C. Surface immunophenotyping was conducted using pre‐titrated antibodies in Brilliant Stain Buffer (BD, 563794) for 15 min at 4°C: Myeloid panel: CD45 (UV395, BioLegend, 103192; 1:100), CD11b (PerCP‐Cy5.5, Invitrogen, 45‐0112‐82; 1:200), F4/80 (PE, BioLegend, 111604; 1:200), CD206 (APC, BioLegend, 141708; 1:100), CD86 (APC/Cy7, BioLegend, 105030; 1:100), MHC‐II (FITC, BioLegend, 107605; 1:100); Lymphocyte panel: CD45 (UV395, BioLegend, 103192; 1:100), CD3 (Pacific Blue, BioLegend, 100214; 1:200), CD4 (PerCP‐Cy5.5, BioLegend, 116012; 1:200), CD8a (APC/Cy7, BioLegend, 100714; 1:200), PD‐1 (PE, BioLegend, 135205; 1:100), Tim‐3 (APC, BioLegend, 119706; 1:100). Stained cells were acquired on a BD FACSAria II. Data analysis employed FlowJo (v10.3.0) software.

### 4.17 Immunohistochemistry (IHC) and Immunofluorescence (IF)

Paraffin‐embedded sections were deparaffinized in xylene and rehydrated through graded ethanol. Antigen retrieval was performed in citrate buffer (110°C, 10 min). Sections were blocked with 5% BSA in PBS for 1 h at room temperature and incubated with primary antibodies: Cacpase3 (CST, 9662; 1:1000), C3(Santa Cruz Biotechnology, sc‐28294; 1:100), C3aR (Santa Cruz Biotechnology, sc‐133172; 1:200), CD68(Abcam, ab303565; 1:200) overnight at 4°C. After washing with PBS‐T (0.1% Tween‐20), IF samples were probed with secondary antibodies for 1 h at room temperature: IF: Goat Anti‐Mouse IgG H&L (Alexa Fluor 488, Abcam, ab150113; 1:800), Goat Anti‐Rabbit IgG H&L (Alexa Fluor 488, Abcam, ab150077; 1:800), Goat Anti‐Rabbit IgG H&L (Alexa Fluor 555, Abcam, ab150078; 1:800); IHC: Goat Anti‐Rabbit IgG H&L (HRP, Abcam, ab6721; 1:1000). Nuclei were counterstained with DAPI for IF or hematoxylin for IHC. IF slides were mounted with Antifade Mounting Medium (Beyotime, P0126), and IHC sections were dehydrated and mounted with Neutral Balsam. Imaging was performed using a Leica fluorescence microscope and analyzed using a LAS X (3.7.6) software. Quantification of immunofluorescence (IF) and immunohistochemistry (IHC) images was performed using Fiji (ImageJ) software.

### 4.18 Real‐Time PCR

Total RNA was isolated from tumor tissues using TRIzol Reagent (Invitrogen, 15596026CN) and reverse‐transcribed into cDNA with the Hifair II 1st Strand cDNA Synthesis SuperMix (Yeasen, 11120ES60). Quantitative PCR amplification was performed on a CFX Connect Real‐Time System (Bio‐Rad, USA) under the following conditions: 95°C for 5 min, followed by 40 cycles of 95°C for 10 s and 60°C for 30 s. Gene expression was quantified via the 2−ΔΔCT method using GAPDH (Forward: 5′‐TTTGCGTCGCCAGCCG‐3′; Reverse: 5′‐CCGTTCTCAGCCTTGACGGT‐3′) as the endogenous control. Target primers included C3 (Forward: 5′‐CTGCCCAGTTTCGAGGTCAT‐3′; Reverse: 5′‐CAATCGGAATGCGCTTGAGG‐3′). All the primers were synthesized by Sangon Biotech Co., Ltd (Shanghai, China).

### 4.19 Development of CCRTIM Model

The predictive model was developed through systematic integration of single‐cell derived immune signatures and bulk transcriptomic profiling. Twenty‐five immune features identified from single‐cell RNA sequencing were quantified across the In‐House cohort (post‐RT samples with prognostic information, *n* = 57) and publicly available TCGA‐CESC(RT) cohort (patients receiving RT, *n* = 187) using Gene Set Variation Analysis (GSVA) with the GSVA (v1.50.5) R package. Cohort compatibility was verified through principal component analysis (PCA) and Uniform Manifold Approximation and Projection (UMAP), enabling dataset merging (*n* = 244) followed by stratified random splitting into training (80%, *n* = 195) and validation (20%, *n* = 49) subsets. To enhance the efficiency, interpretability, and generalization ability of the model, we perform feature selection to identify a subset of the original features that are most valuable for model prediction. Feature selection employed SHapley Additive exPlanations (SHAP) with the Python package shap (v0.47.2) to retain variables with importance scores >0.2 (15 features), subsequently refined via Pearson correlation filtering with the Python package scipy (v1.15.2) (|r| <0.7) to eliminate multicollinearity, yielding eight non‐redundant predictors. Fifteen machine learning algorithms—including tree‐based methods (Decision Tree, Random Forest), boosting variants (Gradient Boosting, AdaBoost, HGBoost, XGBoost, CatBoost), linear models (Logistic Regression), kernel methods (SVM), instance‐based learners (k‐Nearest Neighbors), probabilistic approaches (Gaussian Process), and neural networks with different numbers of hidden layers and nodes (MLP1‐4)—implemented using the scikit‐learn package in Python were evaluated through grid search optimization (100 iterations/algorithm) to classify patient overall survival (OS) with 5‐fold cross‐validation. Hyperparameter tuning maximized area under the ROC curve (AUC), with final model selection based on median performance across validation folds. The optimized multilayer perceptron (MLP3, ReLU activation) demonstrated superior prognostic stratification (AUC = 0.762 ± 0.004). Experiments were conducted on an NVIDIA RTX 3090 GPU and an Intel CPU i9‐10900K@3.7 GHz.

### 4.20 Statistical Analysis

Data are presented as mean ± SEM. All statistical analyses were performed using GraphPad Prism 8 software or R (version 4.3.0). For comparisons between two groups, paired or unpaired two‐tailed Student's *t*‐tests, or the non‐parametric Wilcoxon rank‐sum test, were applied. For comparisons among multiple groups, one‐way or two‐way analysis of variance (ANOVA) was conducted as appropriate. Where multiple pairwise comparisons were performed, *p*‐values were adjusted using the Benjamini–Hochberg (FDR) correction. Survival curves were compared using the log‐rank (Mantel–Cox) test. Significance levels are denoted as follows: ^*^ *p* <0.05, ^**^ *p* <0.01, ^***^ *p* <0.001, ^****^ *p* <0.0001; ns indicates non‐significant (*p* >0.05).

## Author Contributions

Y.Z. and H.C. conceived and supervised the project. L.W. and F.Z. performed the data analyses. L.W., J.Z., Z.D., Z.X., and X.L. designed and performed the research. H.C., J.Z., and X.L. collected and provided the human specimens. Y.Z., H.C., L.W., and J.Z. interpreted the results. Y.Z., H.C., and L.W. wrote the original manuscript with input from all the other authors. L.W. and X.L. revised the manuscript and contributed additional statistical analyses. All authors read and approved the final version of the submitted manuscript.

## Funding

The study is supported by funds from the National Key Research and Development Program of China (2023YFC1404100, 2023YFC1404102 to Y.Z.; 2023YFA1800700, 2023YFA1800702 to Y.Z.), the National Natural Science Foundation of China (81972670 to Y.Z. and 81903129 to H.C.), the Fundamental Research Funds for the Central Universities (YG2023ZD06 to Y.Z.), the Shanghai Shenkang Hospital Development Center (SHDC12024121 to X.L.), and the Jiebanguashuai project of Renji Hospital (RJTJ25‐MS‐002 to H.C.).

## Consent

All the authors have signed the consent form for publication.

## Conflicts of Interest

The authors declare no conflicts of interest.

## Supporting information




**Supporting File**: advs73754‐sup‐0001‐SuppMat.docx.

## Data Availability

The data that support the findings of this study are available from the corresponding author upon reasonable request.
