# Supplementary material for: Immune Predictors of Radiotherapy Outcomes in Cervical Cancer
Source: Adv Sci (Weinh). 2026 Jan 21;13(16):e09784. doi: 10.1002/advs.202509784 (PMC13042687; doi:10.1002/advs.202509784)
Supplement: Supplementary file 1 — Supporting File: advs73754‐sup‐0001‐SuppMat.docx. [file ADVS-13-e09784-s001.docx]

**Supporting Information**

**
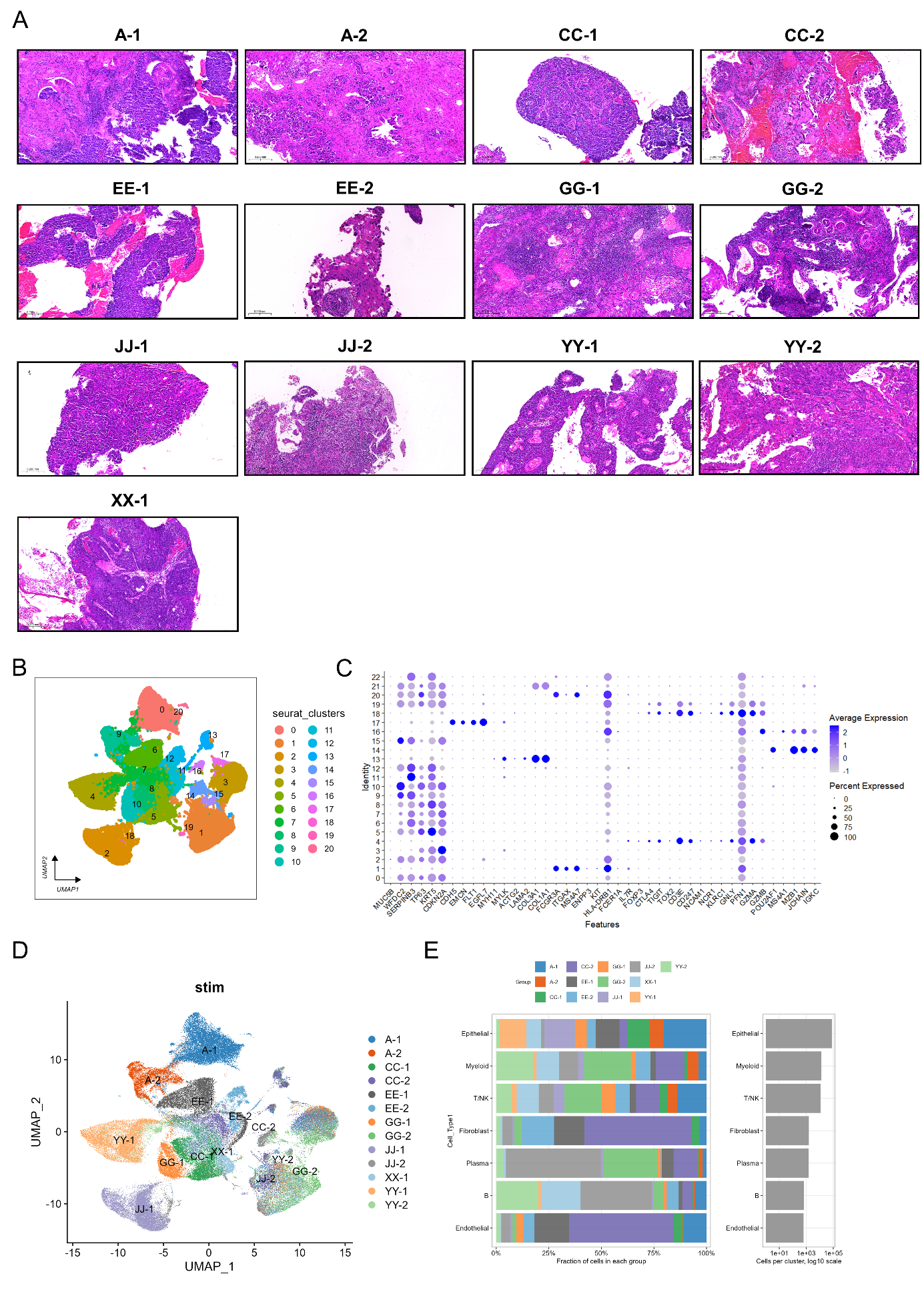
** **Figure S1.** Annotation and distribution of cellular subsets in cervical cancer. **A.** Representative H&E staining of tumor specimens used for scRNA-seq. **B.** Integrated UMAP visualization of 23 transcriptionally heterogeneous cell clusters across 13 tumor specimens (pre-RT n=7; post-RT n=6). **C.** Dot plot displaying cluster-specific canonical marker expression levels (color intensity) and detection frequencies (dot size). **D.** UMAP projection stratified by sample origin. **E.** Dual-axis bar plot illustrating comparative analysis of cell type proportions (left x-axis, %) and absolute cellular abundances (right x-axis) across sample origin.


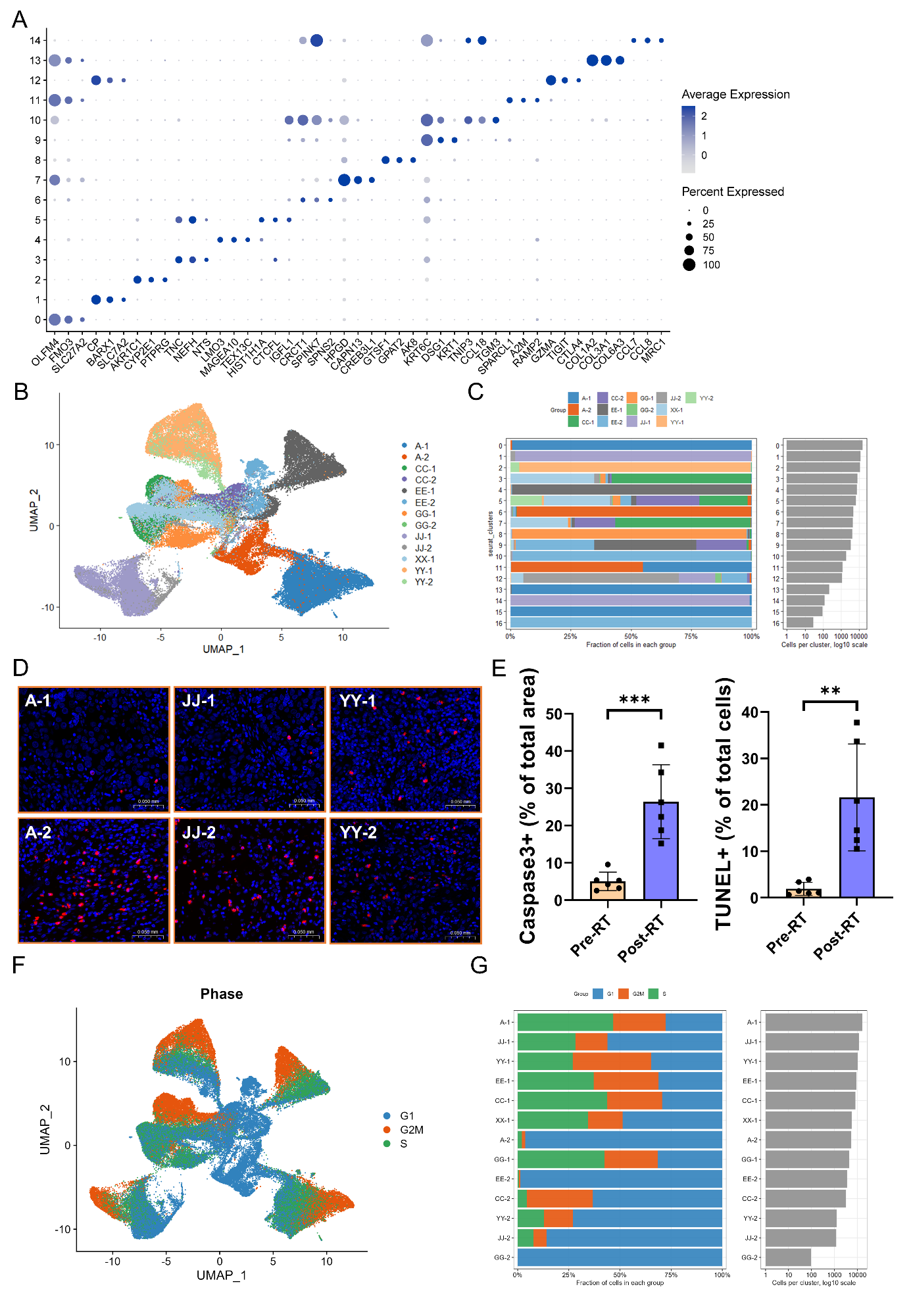


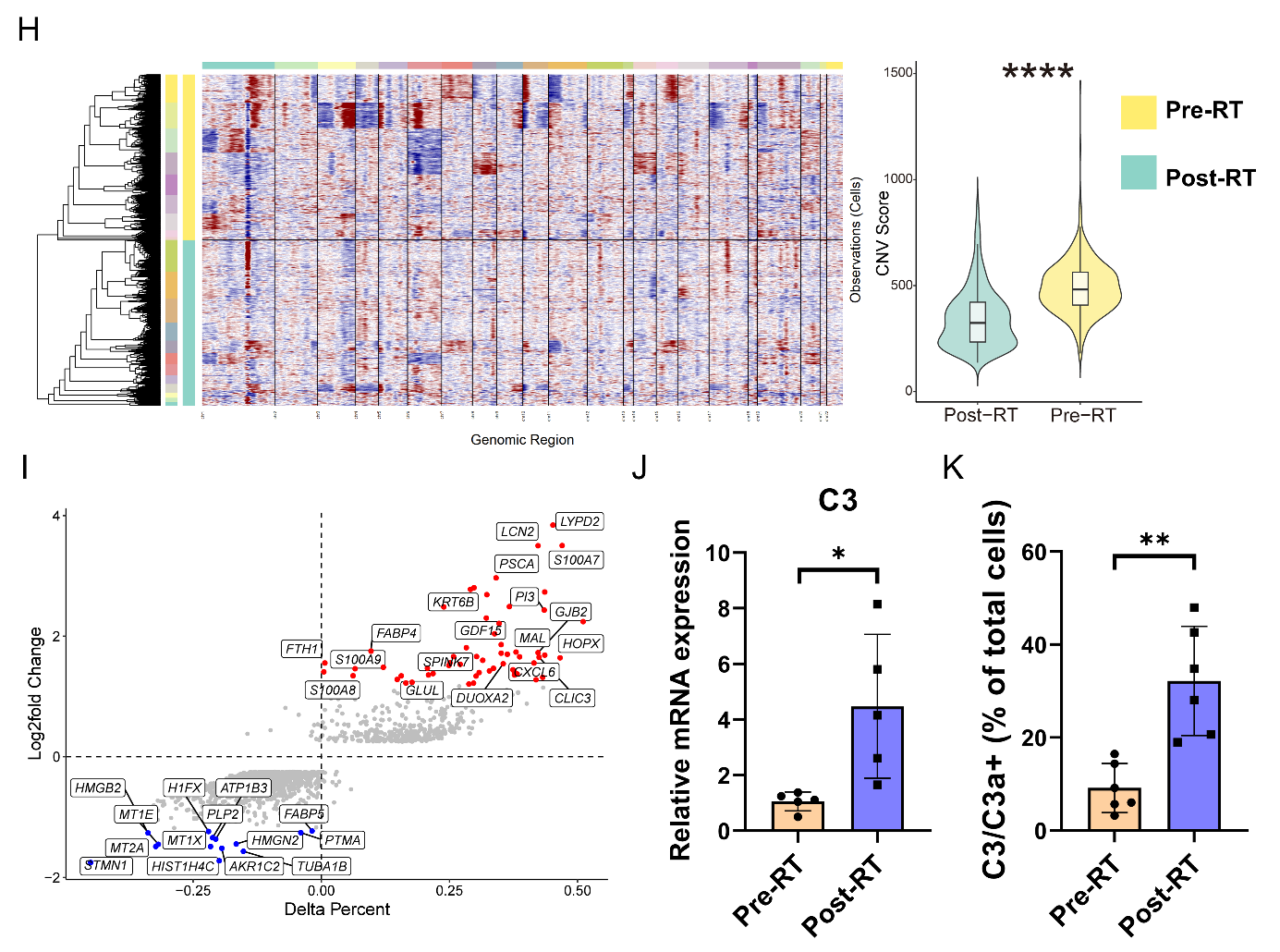


**Figure S2**. Radiotherapy induces epithelial cell cycle arrest and reduces malignant potential. **A.** Dot plot displaying cluster-defining marker gene expression levels (color intensity) and detection frequencies (dot size). **B.** UMAP visualization of epithelial cells stratified by sample origin. **C.** Dual-axis bar plot quantifying epithelial cell proportions (left x-axis, %) and absolute counts (right x-axis) across sample origin. **D.** Representative TUNEL staining images of the samples used for single‑cell RNA sequencing. **E.** Quantitative analysis of Caspase-3 (left) and TUNEL (right) staining in paired pre-RT/post-RT tumor tissues. **F.** Cell cycle phase mapping: UMAP projection of epithelial cells annotated by cell cycle phases (G1/S/G2M). **G.** Dual-axis bar plot comparing cell cycle phase cell proportions (left x-axis, %) and absolute counts (right x-axis) between pre-RT and post-RT conditions. **H.** Copy number variation (CNV) analysis: Composite visualization with CNV pattern heatmap (left) and CNV score violin plot (right). **I.** Volcano plot of differentially expressed genes in epithelial cells post-RT versus pre-RT. **J.** Relative C3 mRNA levels in paired pre-RT/post-RT tumor tissues. **K.** Quantitative analysis of complement C3/C3a staining in paired pre-RT/post-RT tumor tissues. Data presented as mean ± SEM. *p < 0.05, **p < 0.01, ***p < 0.001, ****p < 0.0001. Statistical analysis was performed using the Wilcoxon rank-sum test (H), and two-tailed Student’s t-test (E, J and K).


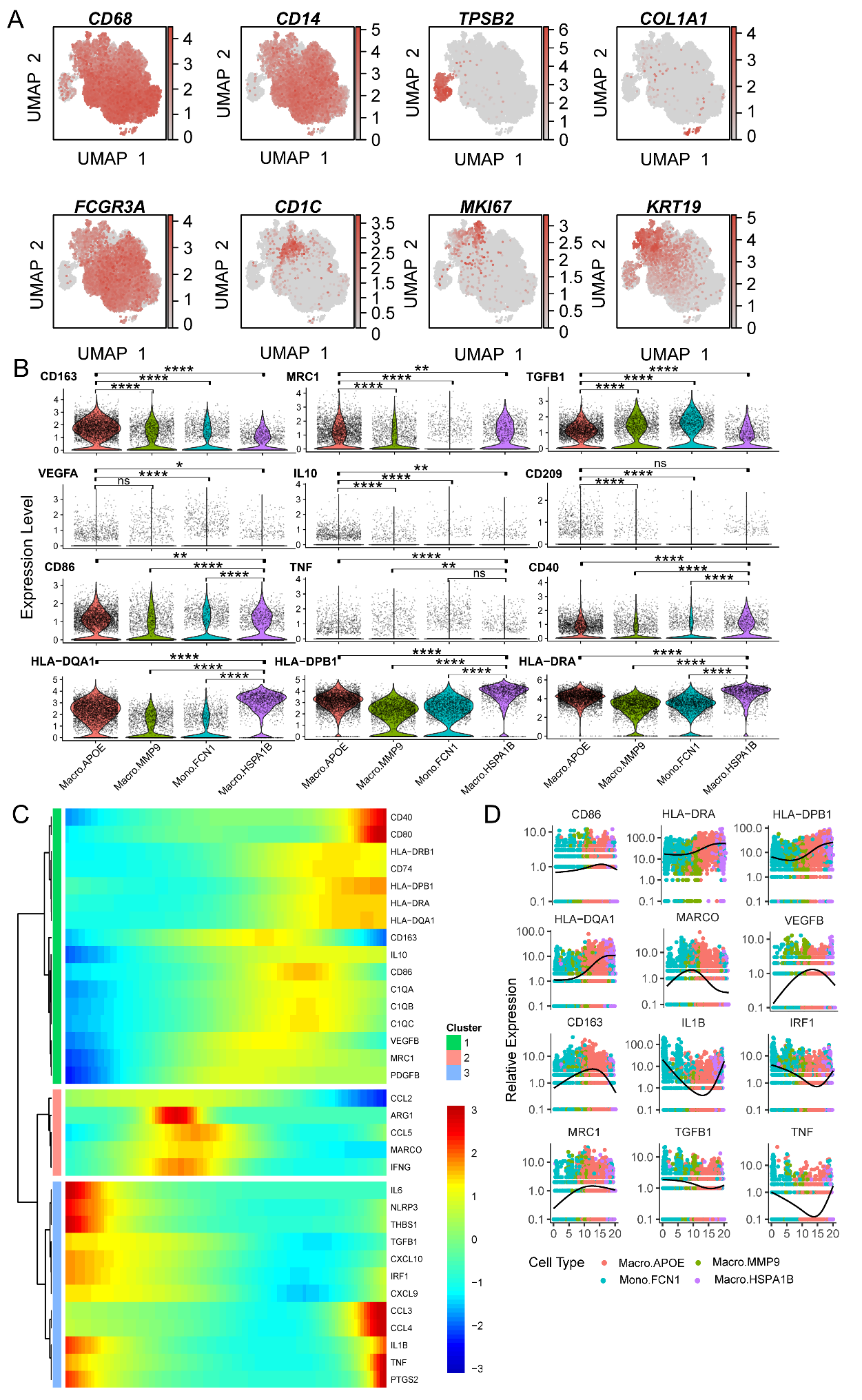


**Figure S3**. Radiotherapy promotes macrophage infiltration and enhances their antigen presentation capabilities. **A.** Feature plot displaying the expression of marker genes in myeloid cells. **B.** Violin plot showing expression of macrophage-related genes in macrophage and monocyte clusters. **C.** Heatmap of macrophage-related gene expression along pseudotime. **D.** Temporal expression patterns of key macrophage-related gene. *p < 0.05, **p < 0.01, ***p < 0.001, ****p < 0.0001, ns, non-signiﬁcant, p > 0.05. Statistical analysis was performed using the Wilcoxon rank-sum test (B).

**
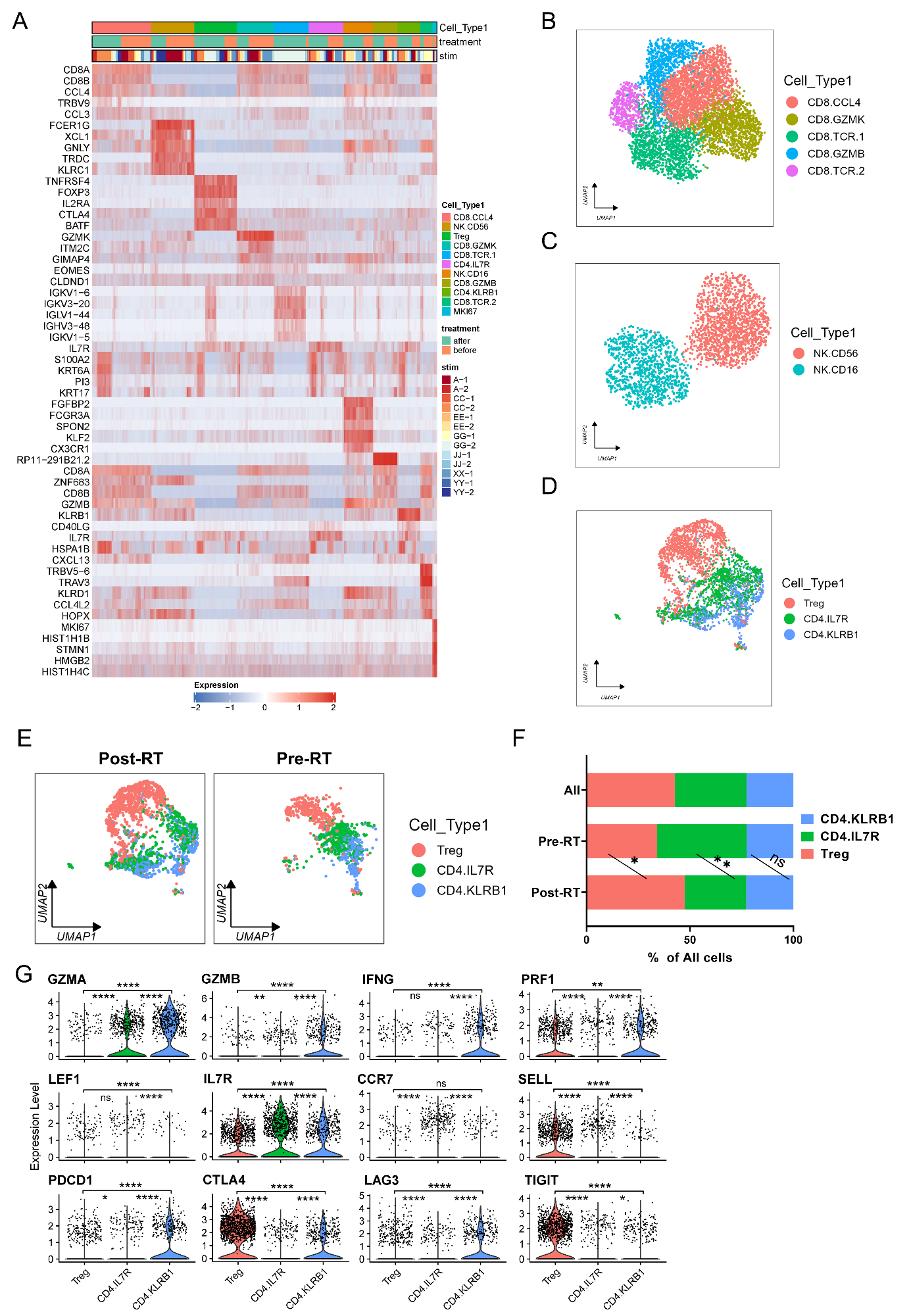
Figure S4.** Radiation-driven remodeling of CD4+ T cell states. **A.** Heatmap of top 5 marker genes per cluster. **B.** Integrated UMAP of 5 transcriptionally distinct CD8+ T cell. **C.** Integrated UMAP of 2 NK cell clusters. **D.** Integrated UMAP of 3 CD4+T cell clusters. **E.** UMAP visualization of CD4+ T cell composition stratified by radiotherapy status (post-RT vs pre-RT). **F.** Stacked bar plot depicting proportional changes in CD4+ T cell populations between pre-RT and post-RT conditions. **G.** Violin plot showing expression of key T cell-related genes in CD4+ T cell clusters. *p < 0.05, **p < 0.01, ***p < 0.001, ****p < 0.0001, ns, non-signiﬁcant, p > 0.05. Statistical analysis was performed using the Wilcoxon rank-sum test (F and G).

**
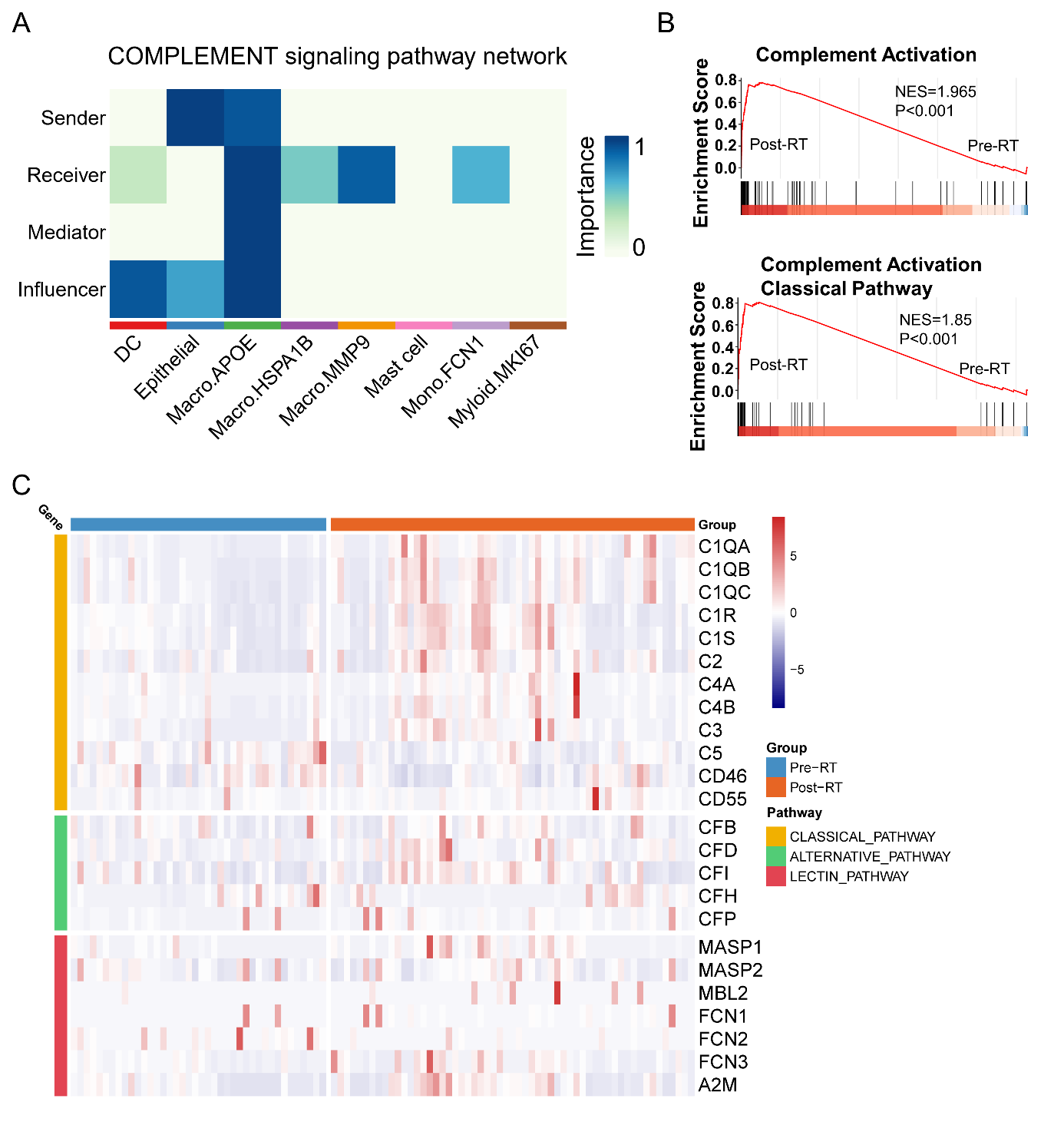
Figure S5.** Radiotherapy enhances the crosstalk of epithelial and macrophage cells via complement pathway. **A.** Heatmap of network centrality measures for the complement signaling pathway. **B.** GSEA results demonstrating significant enrichment of complement activation and the classical complement pathway post-radiotherapy. **C.** Heatmap showing the genes expression levels of three complement activation pathways in bulk RNA sequencing data pre-RT and post-RT.

**
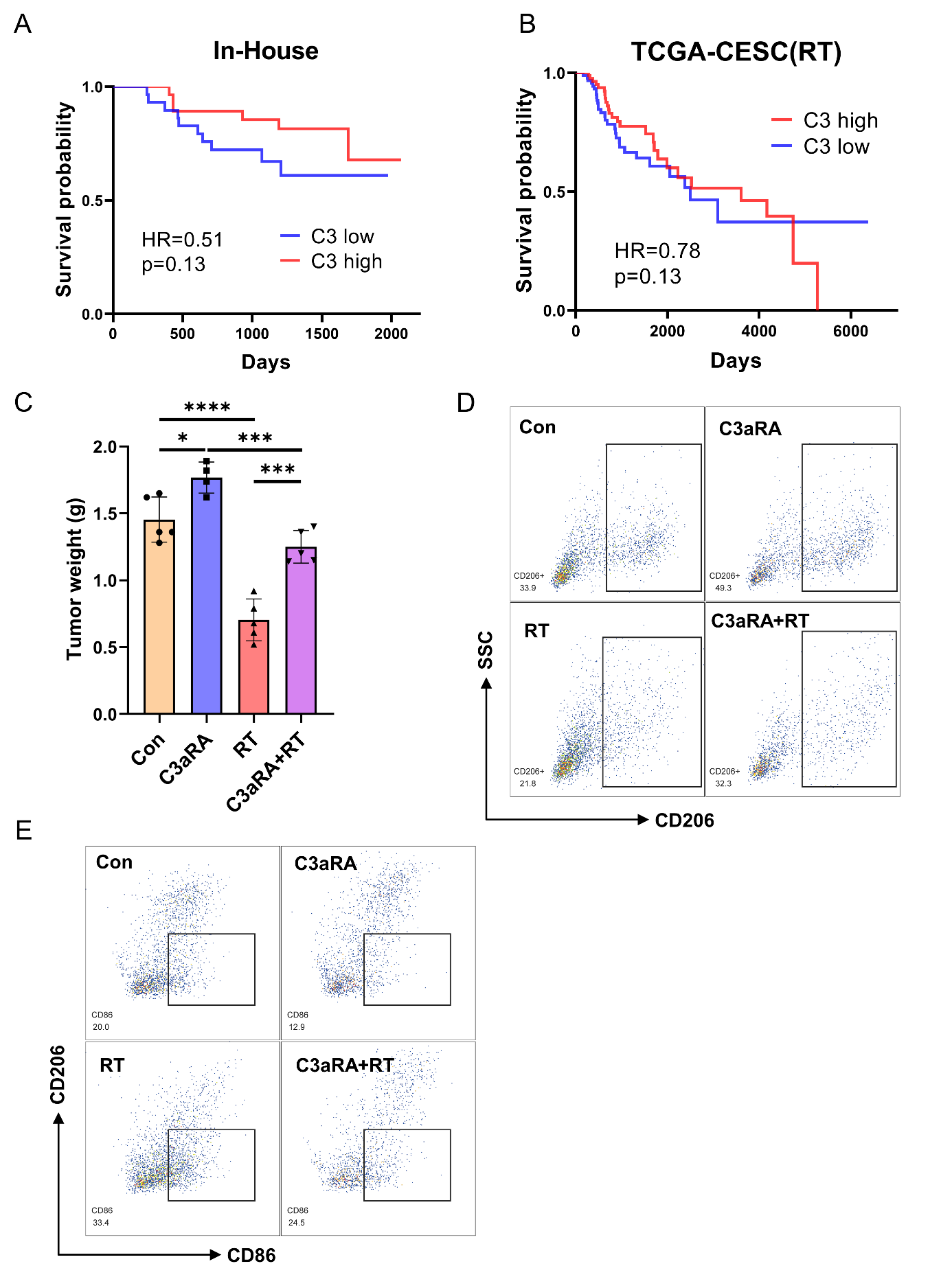
** **Figure S6.** Blocking the complement C3a receptor impairs the efficacy of radiotherapy in tumor control. **A.** Kaplan–Meier plot showing overall survival stratified by C3 expression from In-House cohort (n=57) following RT. **B.** Kaplan–Meier plot showing overall survival stratified by C3 expression from TCGA-CESC(RT) cohort (n=187) following RT. **C.** Tumor weight at the experimental endpoint across different treatment conditions. n=5 per group. **D.** Representative flow cytometry dot plots of CD206+ macrophages’ frequencies in mice tumor. **E.** Representative flow cytometry dot plots of CD86+ macrophages’ frequencies in mice tumor. Data presented as mean ± SEM.*p < 0.05, **p < 0.01, ***p < 0.001, ****p < 0.0001. Statistical analysis was performed using log-rank (Mantel–Cox) test (A and B), and one-way ANOVA with Tukey’s test (C).


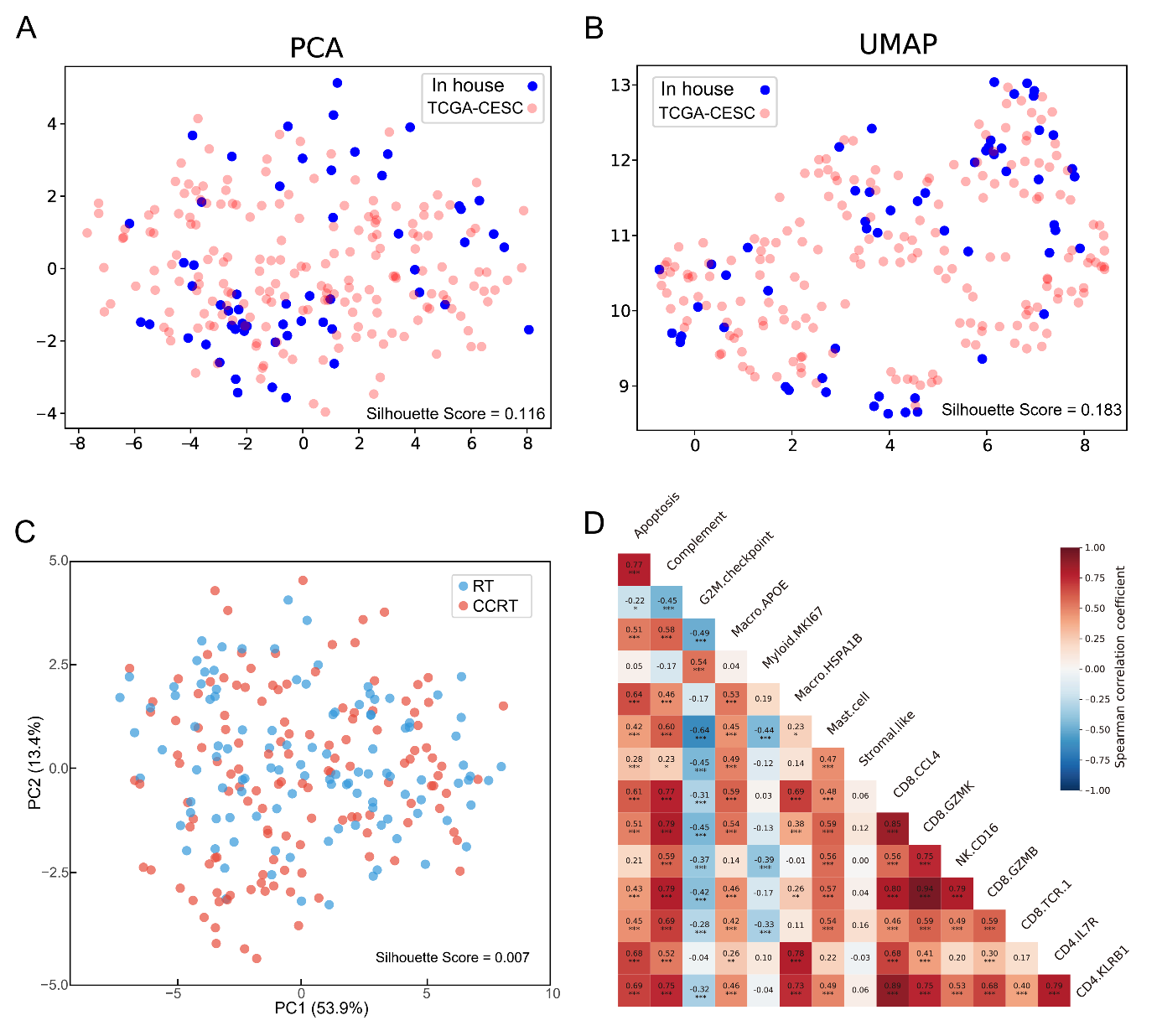
 **Figure S7.** Developing CCRTIM model integrated scRNA-seq and bulk-seq predicts radiotherapy outcomes in cervical cancer. **A.** Principal Component Analysis (PCA) plots demonstrating concordant feature distribution between In-House cohort (n=57) and TCGA-CESC(RT) cohort (n=187). **B.** Uniform Manifold Approximation Projection (UMAP) plots demonstrating concordant feature distribution between In-House cohort (n=57) and TCGA-CESC(RT) cohort (n=187). **C.** Principal Component Analysis (PCA) plots demonstrating concordant feature distribution between samples recived radiotherapy-alone (RT, n=112) and concurrent chemoradiotherapy (CCRT, n=132). **D.** Pearson correlation heatmap of 15 SHAP-selected features.

**Table S1. Patient information of Bulk-seq samples**

| **Patient ID** | **Age** | **FIGO Stage** | **CCRT** | **Status at last follow-up** | **Follow-up time (D)** | **Sample**  **name** | **Sample time** |
| --- | --- | --- | --- | --- | --- | --- | --- |
| 1 | 63 | Ⅳa | N | D | 243 | 1-2 | Post-RT |
| 2 | 49 | IIIc1 | Y | D | 433 | 2-2 | Post-RT |
| 3 | 66 | Ⅳa | Y | A | 829 | 3-2 | Post-RT |
| 4 | 48 | IIIc1 | Y | A | 1482 | 4-2 | Post-RT |
| 5 | 33 | IIIc1 | Y | A | 857 | 5-1 | Pre-RT |
|  |  |  |  |  |  | 5-2 | Post-RT |
| 6 | 65 | IIIc1 | Y | A | 932 | 6-2 | Post-RT |
| 7 | 82 | IIb | N | A | 1742 | 7-1 | Pre-RT |
|  |  |  |  |  |  | 7-2 | Post-RT |
| 8 | 65 | IIIc2 | Y | A | 1949 | 8-2 | Post-RT |
| 9 | 50 | Ⅳa | N | D | 254 | 9-2 | Post-RT |
| 10 | 48 | IIIc1 | Y | D | 371 | 10-2 | Post-RT |
| 11 | 75 | IIIc1 | Y | A | 1258 | 11-2 | Post-RT |
| 12 | 57 | IIb | Y | A | 1689 | 12-2 | Post-RT |
| 13 | 62 | IIIc1 | Y | A | 997 | 13-2 | Post-RT |
| 14 | 66 | Ⅳa | Y | A | 1306 | 14-1 | Pre-RT |
|  |  |  |  |  |  | 14-2 | Post-RT |
| 15 | 54 | IIb | N | D | 466 | 15-1 | Pre-RT |
|  |  |  |  |  |  | 15-2 | Post-RT |
| 16 | 65 | IIb | Y | D | 610 | 16-2 | Post-RT |
| 17 | 73 | IIIc1 | Y | D | 1324 | 17-1 | Pre-RT |
|  |  |  |  |  |  | 17-2 | Post-RT |
| 18 | 65 | IIIc2 | Y | A | 997 | 18-1 | Pre-RT |
|  |  |  |  |  |  | 18-2 | Post-RT |
| 19 | 55 | Ⅳa | Y | D | 432 | 19-2 | Post-RT |
| 20 | 54 | IIIc2 | Y | A | 995 | 20-1 | Pre-RT |
|  |  |  |  |  |  | 20-2 | Post-RT |
| 21 | 69 | IIb | Y | A | 1687 | 21-1 | Pre-RT |
|  |  |  |  |  |  | 21-2 | Post-RT |
| 22 | 52 | IIIc1 | Y | A | 902 | 22-1 | Pre-RT |
|  |  |  |  |  |  | 22-2 | Post-RT |
| 23 | 53 | IIb | Y | A | 1974 | 23-1 | Pre-RT |
|  |  |  |  |  |  | 23-2 | Post-RT |
| 24 | 63 | IIIc2 | Y | A | 1911 | 24-2 | Post-RT |
| 25 | 65 | IIIc2 | Y | D | 1206 | 25-2 | Post-RT |
| 26 | 59 | IIIc1 | Y | D | 1070 | 26-2 | Post-RT |
| 27 | 66 | IIb | Y | A | 1285 | 27-2 | Post-RT |
| 28 | 52 | IIIc2 | Y | A | 1602 | 28-2 | Post-RT |
| 29 | 69 | Ⅳa | Y | A | 1077 | 29-1 | Pre-RT |
|  |  |  |  |  |  | 29-2 | Post-RT |
| 30 | 53 | IIIc1 | Y | A | 1453 | 30-1 | Pre-RT |
|  |  |  |  |  |  | 30-2 | Post-RT |
| 31 | 47 | IIIc2 | Y | A | 1769 | 31-1 | Pre-RT |
|  |  |  |  |  |  | 31-2 | Post-RT |
| 32 | 68 | IIIa | Y | A | 673 | 32-1 | Pre-RT |
|  |  |  |  |  |  | 32-2 | Post-RT |
| 33 | 60 | IIIc2 | N | D | 472 | 33-1 | Pre-RT |
|  |  |  |  |  |  | 33-2 | Post-RT |
| 34 | 52 | IIIc2 | Y | A | 1738 | 34-1 | Pre-RT |
|  |  |  |  |  |  | 34-2 | Post-RT |
| 35 | 48 | IIIc1 | Y | D | 931 | 35-1 | Pre-RT |
|  |  |  |  |  |  | 35-2 | Post-RT |
| 36 | 32 | IIIc2 | Y | D | 643 | 36-1 | Pre-RT |
|  |  |  |  |  |  | 36-2 | Post-RT |
| 37 | 35 | IIIc2 | Y | A | 888 | 37-2 | Post-RT |
| 38 | 55 | Ⅳa | Y | A | 1664 | 38-1 | Pre -RT |
|  |  |  |  |  |  | 38-2 | Post -RT |
| 39 | 68 | IIb | N | A | 1427 | 39-1 | Pre-RT |
|  |  |  |  |  |  | 39-2 | Post- RT |
| 40 | 64 | IIb | Y | A | 1500 | 40-2 | Post-RT |
| 41 | 66 | IIIc1 | N | D | 404 | 41-1 | Pre-RT |
|  |  |  |  |  |  | 41-2 | Post-RT |
| 42 | 61 | IIIc2 | N | A | 1215 | 42-1 | Pre-RT |
|  |  |  |  |  |  | 42-2 | Post-RT |
| 43 | 51 | Ⅳa | Y | A | 1655 | 43-1 | Pre-RT |
|  |  |  |  |  |  | 43-2 | Post-RT |
| 44 | 78 | Ⅳa | Y | A | 1069 | 44-1 | Pre-RT |
|  |  |  |  |  |  | 44-2 | Post-RT |
| 45 | 56 | Ⅳa | Y | A | 1230 | 45-1 | Pre-RT |
|  |  |  |  |  |  | 45-2 | Post-RT |
| 46 | 60 | IIIc1 | Y | A | 1082 | 46-1 | Pre-RT |
|  |  |  |  |  |  | 46-2 | Post-RT |
| 47 | 59 | IIIc1 | Y | A | 1394 | 47-2 | Post-RT |
| 48 | 54 | IIIc1 | Y | A | 2068 | 48-1 | Pre-RT |
|  |  |  |  |  |  | 48-2 | Post- RT |
| 49 | 56 | IIIc1 | Y | A | 1472 | 49-1 | Post-RT |
| 50 | 54 | Ⅳa | Y | D | 1191 | 50-1 | Pre-RT |
|  |  |  |  |  |  | 50-2 | Post-RT |
| 51 | 54 | IIb | Y | D | 708 | 51-2 | Post-RT |
| 52 | 61 | IIIc1 | Y | A | 1667 | 52-1 | Pre-RT |
|  |  |  |  |  |  | 52-2 | Post-RT |
| 53 | 63 | IIIa | Y | A | 1250 | 53-1 | Pre-RT |
|  |  |  |  |  |  | 53-2 | Post-RT |
| 54 | 46 | IIIc1 | Y | A | 1841 | 54-1 | Pre-RT |
|  |  |  |  |  |  | 54-2 | Post-RT |
| 55 | 67 | IIIc1 | Y | A | 1779 | 55-1 | Pre-RT |
|  |  |  |  |  |  | 55-2 | Post-RT |
| 56 |  | IIIc1 | Y | A | 1592 | 56-1 | Pre-RT |
|  |  |  |  |  |  | 56-2 | Post-RT |
| 57 | 68 | IIIc1 | Y | A | 1921 | 57-2 | Post-RT |
| 58 | 50 | IIIc1 | Y | A | 2236 | 58-1 | Pre-RT |
| 59 | 55 | IIIc1 | Y | A | 827 | 59-1 | Pre-RT |
| 60 | 68 | IIIc1 | Y | A | 2240 | 60-1 | Pre-RT |
| 61 | 33 | IIIc1 | Y | A | 2239 | 61-1 | Pre-RT |
| 62 | 60 | IIIc1 | Y | A | 2241 | 62-1 | Pre-RT |
| 63 | 66 | IIIc1 | Y | A | 861 | 63-1 | Pre-RT |
| 64 | 75 | IIIc1 | Y | A | 2253 | 64-1 | Pre-RT |
